# Supplementary material for: Insulin action and resistance are dependent on a GSK3β-FBXW7-ERRα transcriptional axis
Source: Nat Commun. 2022 Apr 19;13:2105. doi: 10.1038/s41467-022-29722-6 (PMC9019090; doi:10.1038/s41467-022-29722-6)

Insulin action and resistance are dependent on a GSK3β-FBXW7-ERRα transcriptional axis in mice

Hui Xia, Charlotte Scholtes, Catherine R. Dufour, Carlo Ouellet, Majid Ghahremani, and Vincent Giguère

Supplementary Information

Supplementary Figures 1-8

Supplementary References

Uncropped immunoblots and gels presented in main Figures

Uncropped histology staining sections presented in main Figures

Uncropped immunoblots and gels presented in Supplementary Figures

Supplementary Fig. 1

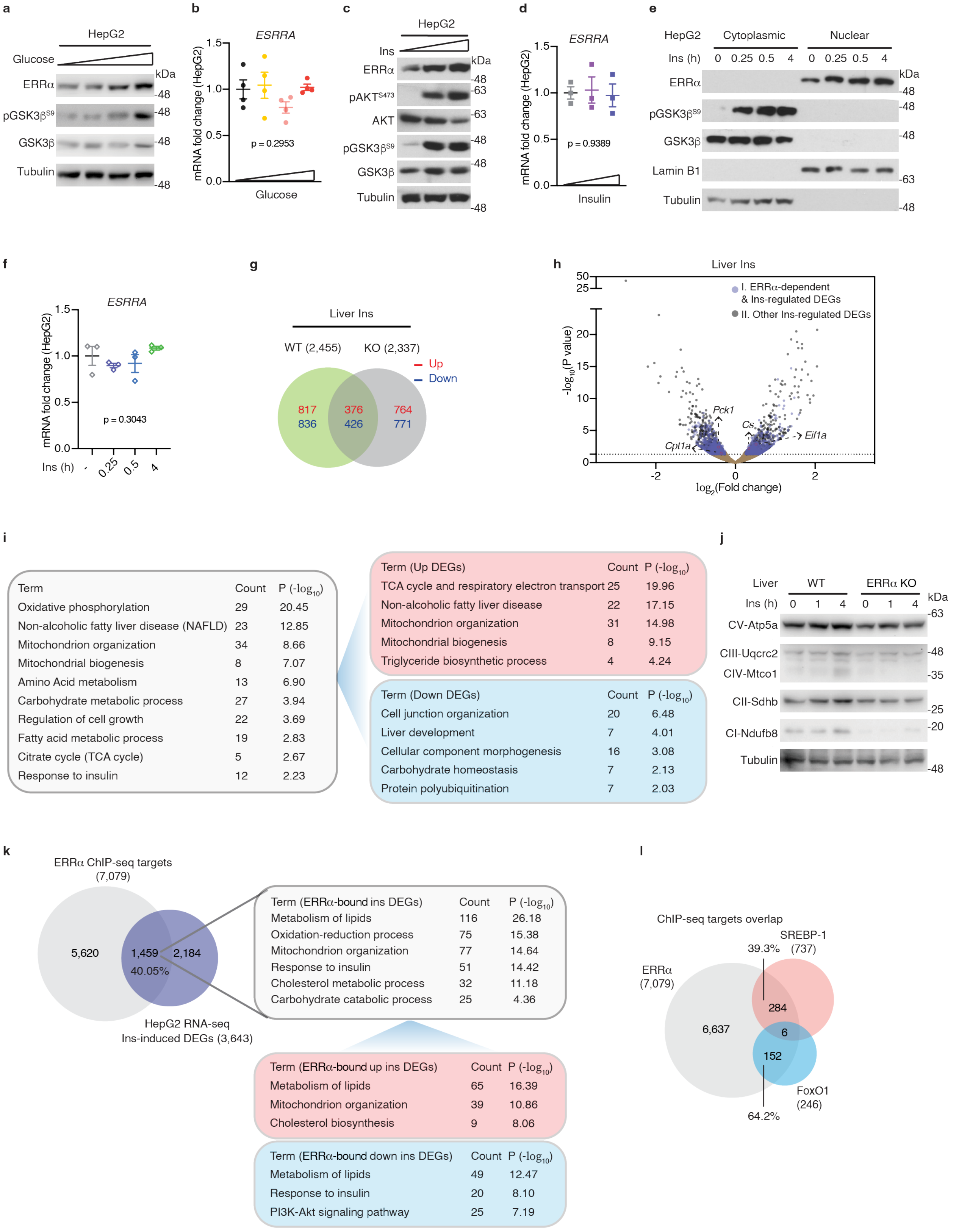

Supplementary Fig. 1 | ERRα is an insulin-stimulated transcription factor. a-d ERRα protein (**a, c**) and mRNA (**b** n = 4 per group**; d** n = 3 per group) levels following glucose or insulin stimulation. **a**, **b** Hepatocytes were cultured in glucose-free media supplemented with 2.5 mM, 5 mM, 12.5 mM and 25 mM D-glucose for 24 h. Media were changed every 6 h. **c**, **d** Hepatocytes were treated with 0, 0.58 μg/ml or 10 μg/ml insulin for 15 min after overnight serum starvation. Cytosolic and nuclear ERRα protein levels (**e**), *ESRRA* mRNA levels (**f,** n = 3 per group) upon 0.58 μg/ml insulin stimulation for the indicated times after overnight serum starvation. **g-I,** Hepatic differentially expressed genes (DEGs) identified in WT and ERRα$^{-/-}$ mice 4 h after insulin administration ($p < 0.05$, $|FC| \geq 1.20$; n = 3 for ERRα KO Ins, n = 4 for the others). **g** Venn diagram of hepatic genes significantly altered by insulin in WT and ERRα$^{-/-}$ mice upon insulin stimulation. **h** Volcano plot of insulin-regulated hepatic genes in WT and ERRα$^{-/-}$ mice. Clusters I and II correspond to genes displayed in the heatmap (Fig. 1m). See also Supplementary Data 1. **i** Metascape[1] pathway analysis of hepatic insulin-regulated genes which were differently expressed between WT and ERRα$^{-/-}$ mice upon insulin. **j** Protein levels of OXPHOS subunits in livers from WT and ERRα-null mice treated with insulin for the indicated times post overnight fasting. Each lane represents liver lysate pooled from three mice. **k** Overlap of insulin-sensitive DEGs[2] (FDR < 0.01) identified in HepG2 cells with ERRα ChIP-seq targets (± 20 kb)[3] and Metascape pathway analysis of ERRα bound insulin-sensitive DEGs. Pathway enrichment of upregulated and downregulated DEGs were shown on the lower panel. **l** Overlap of mouse liver ERRα ChIP-seq targets[3] with SREBP1[4] and FoxO1[5] ChIP-seq targets. Data are presented as means ± SEM, one-way ANOVA (**b**, **d**, **f**). Source data are provided as a Source Data file.

Supplementary Fig. 2

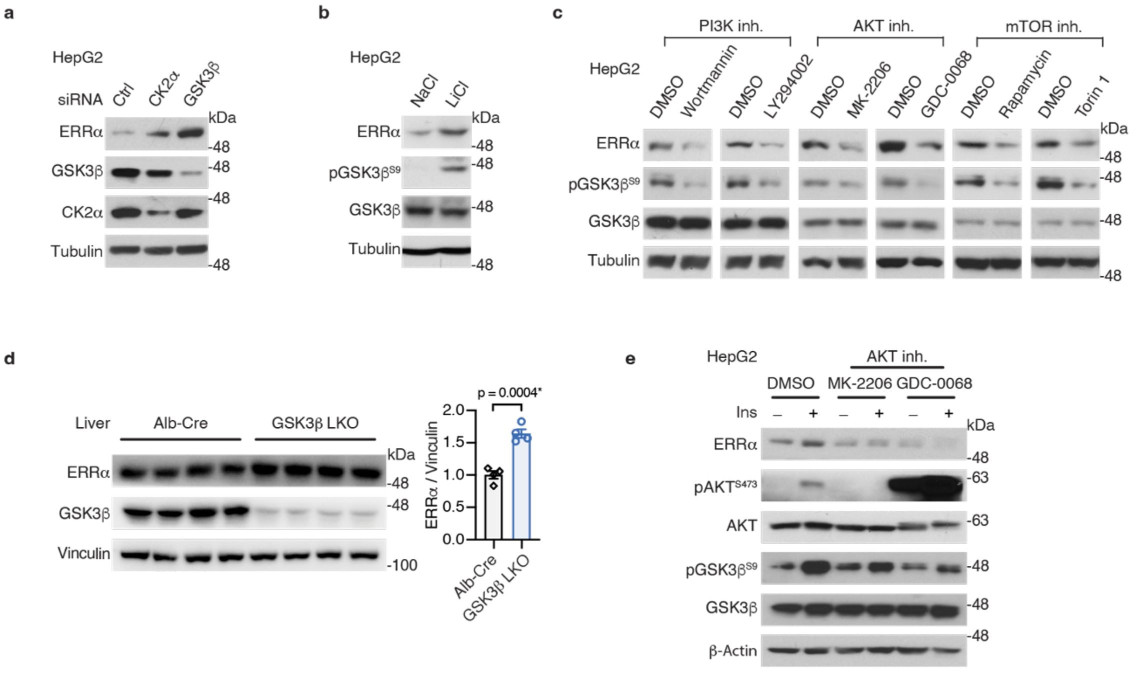

Supplementary Fig. 2 | GSK3β is required for insulin-dependent ERRα stabilization. a ERRα protein levels in HepG2 cells transfected with 20 nM control, CK2α, or GSK3β siRNA for 72 h. **b**, **c** ERRα protein levels in response to pharmacological manipulation of GSK3β activity. Hepatocytes were treated with 40 mM LiCl for 1 h (**b**), 2 μM Wortmannin for 4 h, 10 μM LY294002 for 8 h, 100 nM MK-2206 or GDC-0068 for 4 h, and 100 nM Rapamycin or Torin 1 for 24 h (**c**). **d** Immunoblots (left, each lane represents liver from one mouse, n = 4 per group) and quantification (right) of ERRα protein levels in livers from Alb and GSK3β LKO mice. Quantification data are presented as means ± SEM (*p < 0.05, unpaired two-tailed Student's t test). **e** ERRα protein levels in response to insulin with or without AKT inhibition. Hepatocytes were treated with 100 nM MK-2206 or GDC-0068 for 4 h after overnight serum starvation followed by 10 μg/ml insulin stimulation for another 30 min. Source data are provided as a Source Data file.

Supplementary Fig. 3

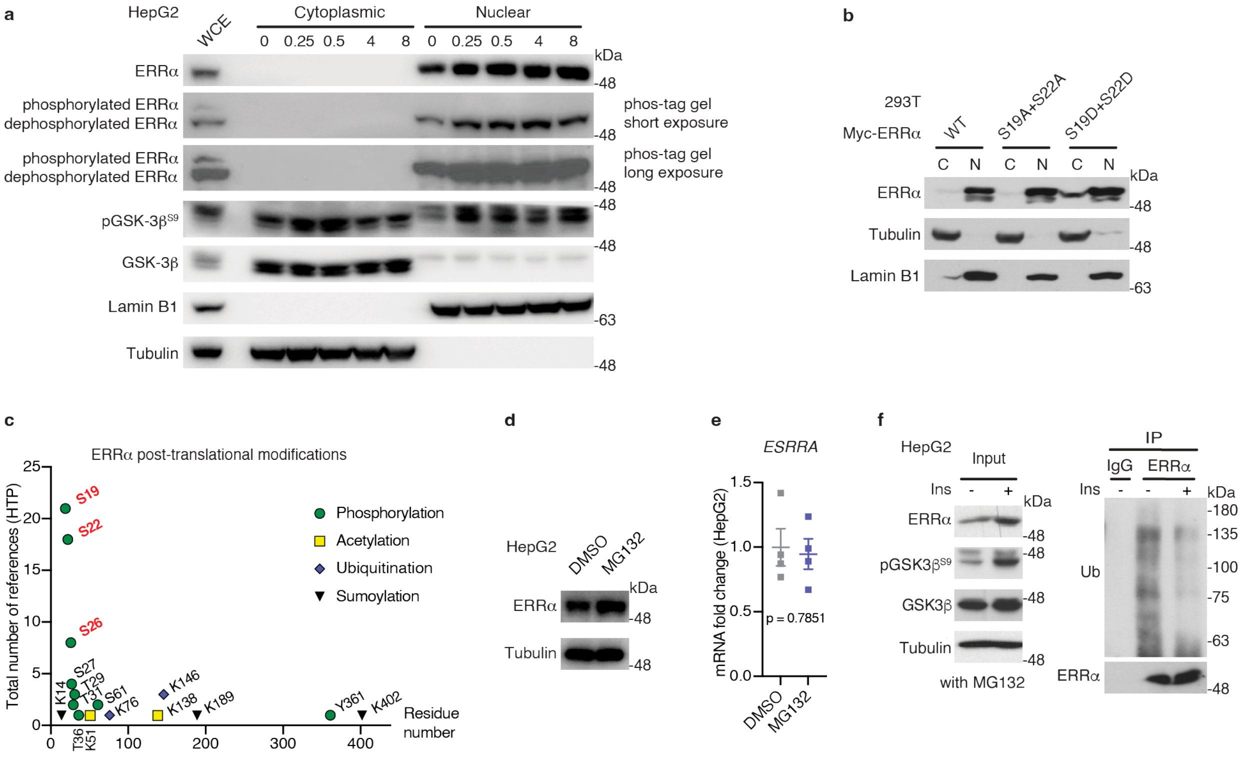

Supplementary Fig. 3 | ERRα phosphorylation by GSK3β leads to its proteasome-mediated degradation. a Examination of ERRα and phosphorylated ERRα protein levels in nuclear extracts from HepG2 cells treated with 0.58 μg/ml insulin for the indicated times after overnight serum starvation. Whole-cell extract (WCE) served as a positive control. **b** ERRα protein levels in the cytoplasmic (C) and nuclear (N) fractions of 293T cells were transfected with ERRα WT, ERRα S19A+S22A, or ERRα S19D+S22D plasmids for 72 h. Cells were treated with MG132 before collection and nuclear extraction. **c** ERRα post-translational modifications revealed by high-throughput papers (HTP) via mass spectrometry (from Phosphositeplus[6] version 6.6.0.4). ERRα protein (**d**) and mRNA (**e)** levels in HepG2 cells treated with MG132. **e** Data are presented as means ± SEM, n = 4 per group, unpaired two-tailed Student's t test. **f** ERRα protein and ubiquitination levels in response to insulin. HepG2 cells were serum starved overnight followed by MG132 treatment for 6 h prior to 10 μg/ml insulin stimulation for another 30 min. Source data are provided as a Source Data file.

Supplementary Fig. 4

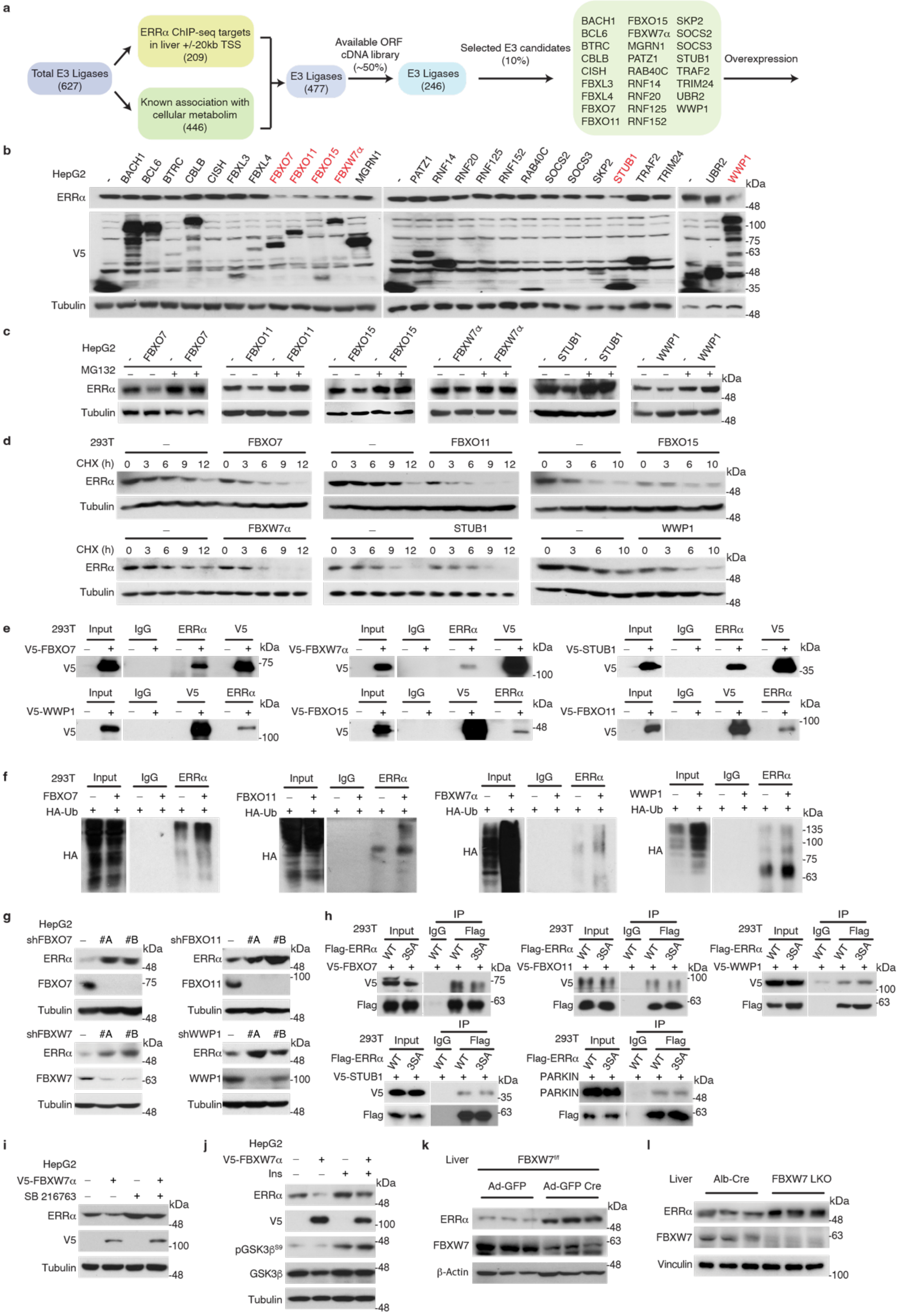

Supplementary Fig. 4 | GSK3β-mediated ERRα degradation is dependent on FBXW7. a Outline of the preliminary screening of the 26 E3 candidates from the total E3 list. See also Supplementary Data 5. **b** ERRα protein levels in HepG2 cells transiently overexpressing the 26 E3 candidates. Six E3 ligases that promoted ERRα degradation were highlighted in red and recognized as potential candidates. **c** HepG2 cells were transiently transfected with the six E3 candidates for 48 h, either DMSO or MG132 were treated prior to sample collection. **d** HEK293T cells were transiently transfected with the six E3 ligase candidates for 36 h prior to treatment with protein synthesis inhibitor cycloheximide (CHX, 100 μg/ml) for the indicated times. **e** Co-IPs were performed using lysates from 293T cells co-transfected with V5-tagged E3 candidates for 24 h. Cells were treated with MG132 prior to harvesting. **f** ERRα ubiquitination levels in 293T cells co-transfected with V5-tagged E3 candidates and HA-Ub for 48 h. Prior to collection, cells were treated with MG132. **g** ERRα protein levels in HepG2 cells following shRNA-mediated knockdown of E3 candidates. **h** Co-IP were performed using lysates from 293T cells transiently co-expressing E3 ligases and Flag-ERRα WT or 3SA mutant. Cells were treated with MG132 prior to collection. **i**, **j** Rescue of FBXW7-mediated ERRα degradation via GSK3β inhibition. HepG2 cells transiently transfected with FBXW7 were treated with 10 μM SB 216763 (GSK3 inhibitor) for 4 h (**i**) or 5 μg/ml insulin for 30 min post overnight serum starvation (**j**). **k** FBXW7$^{f/f}$ mice were injected with $2X10^9$ pfu/mice of GFP or GFP-Cre adenovirus through tail vein and samples were collected 6 days post injection and subjected to immunoblot analysis (each lane represents liver from one mouse, n = 3 per group). **l** ERRα protein levels in livers from Alb and FBXW7 LKO mice. Each lane represents liver from one mouse, n = 3 per group. Source data are provided as a Source Data file.

Supplementary Fig. 5

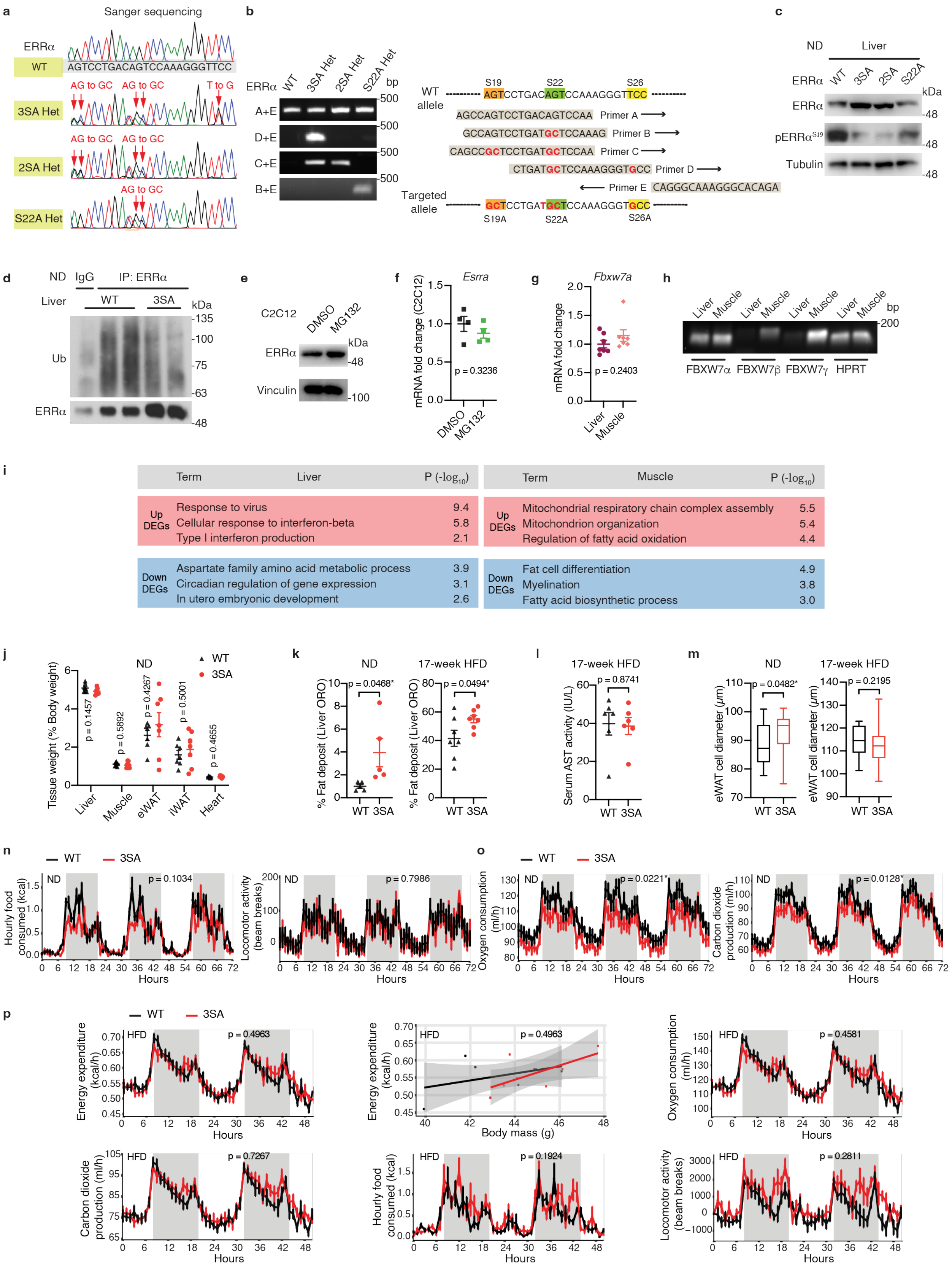

Supplementary Fig. 5 | ERRα^{3SA} mice display transcriptional reprogramming and compromised metabolic homeostasis. **a**, **b** Sanger sequencing (**a**) and PCR genotyping (**b**) of ERRα phospho-mutant and WT mice. **c** Total and S19-phosphorylated ERRα proteins. Each lane represents lysate pooled from four mouse livers. **d** ERRα ubiquitination level. Each lane represents immunoprecipitation performed using liver lysates prepared from two mice. (**e**) ERRα protein and (**f**) mRNA (n = 4 per group) levels. **g** *FBXW7α* mRNA level (n = 7 per group) and distribution of FBXW7 isoform mRNAs (**h**) in C57BL/6N mice. **i** Metascape pathway analysis of the upregulated and downregulated insulin-regulated DEGs identified in ERRα^{3SA} mice. See also Fig. 5d. **j** Percentages of tissue weights (n = 7 per group). eWAT: epididymal white adipose tissue, iWAT: inguinal white adipose tissue. **k** Quantitation of liver ORO from ERRα^{3SA} and WT littermates on a normal diet (ND; n = 5 per group) or high-fat diet (HFD; n = 7 per group). See Fig. 5h for the representative stained sections. **l** Fed serum AST activity under a HFD (n = 6 per group). **m** Quantitation of epididymal white adipocyte sizes, n = 5 per ND-group, n = 6 per HFD-group). Four random sections per mouse were quantified. Box plots show centre line as median, bounds of box as 25th and 75th percentiles, whiskers as minima and maxima. See Fig. 5h for the representative stained sections. **n-p** Indirect calorimetry measurements of ERRα^{3SA} and WT littermates maintained on a ND (**n**, **o** WT, n = 8; 3SA, n = 7) or a 14-week HFD (**p** n = 7 per group). A generalized linear model is fitted to the regression plot of **p**, estimations are displayed by the line, with the standard error shaded in grey. Data are represented as means ± SEM (**f**, **g**, **j**-**l**, **n**, **o**, all other plots except the regression plot of **p**). *$p < 0.05$, unpaired two-tailed Student's t test (**f**, **g**, **j**-**m**), ANOVA (locomotor activity plot of **n** and **p**), and ANCOVA (all other plots of **n** and **p**; **o**). Source data are provided as a Source Data file.

Supplementary Fig. 6

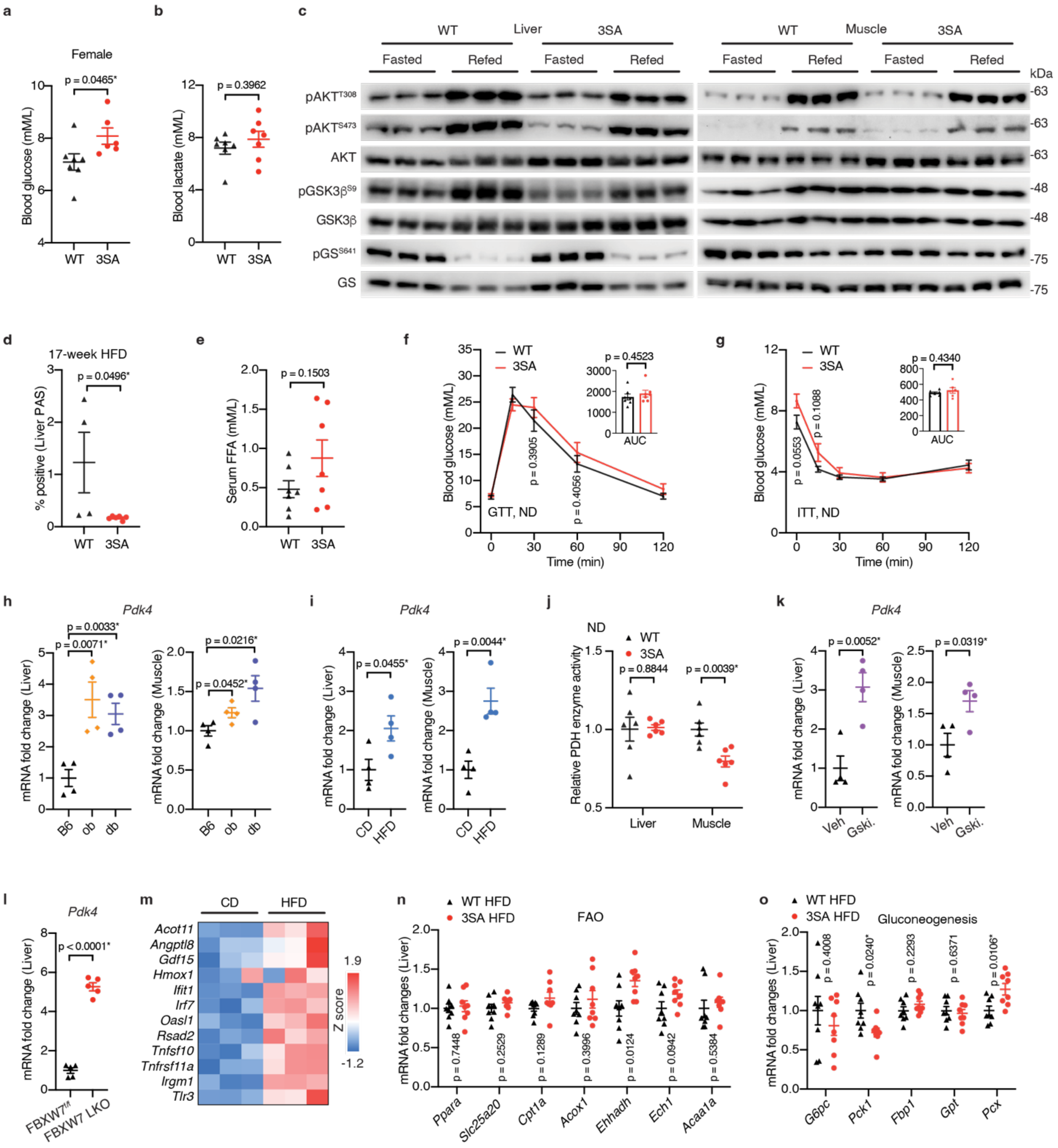

Supplementary Fig. 6 | ERRα^{3SA} phospho-deficient mice develop insulin resistance. a Fed blood glucose concentrations of 3-month-old female ERRα^{3SA} and WT littermates on a ND (WT, n = 7; 3SA, n = 6). **b** Fed blood lactate levels of ND-fed ERRα^{3SA} and WT littermates (n = 7 per group). **c** Immunoblots of the insulin signaling cascade components upon fasting and refeeding used in Fig. 6c. Each lane represents tissue from one mouse, n =3 per group. **d** Quantitation of liver PAS staining of ERRα^{3SA} and WT littermates upon a HFD (WT, n = 4; 3SA, n = 6). See Fig. 6e for the representative stained sections. **e** Fasting serum FFA concentrations under a ND (n = 7 per group). **f** Tolerance test of glucose (GTT) and insulin (ITT) (**g**) were performed at 12 weeks and 10 weeks of age, respectively. Inset graphs show area under the curve (AUC) (**f** n = 7 per group; **g** n = 6 per group). **h** *Pdk4* mRNA levels of control and obese diabetic (ob/ob, db/db) mice (n = 4 per group)**,** and of mice fed either a chow diet (CD) or a HFD for 17 weeks (**i**) (n = 4 per group). **j** Liver and muscle PDH enzyme activity of ND-fed ERRα^{3SA} and WT littermates (n = 6 per group). **k** Liver and muscle *Pdk4* mRNA levels of mice intraperitoneally injected with vehicle or 20 mg/kg GSK3 inhibitor SB216763 for 14 days. Mice were on a CD for 17 weeks before injections (n = 4 per group). **l** *Pdk4* mRNA levels in control and FBXW7-null livers (n = 5 per group). **m** Z score heatmap plotted according to GSE77625[7] to demonstrate the expression of genes shown in Fig. 6n on a CD or HFD. mRNA levels of genes involved in FAO (**n**) and gluconeogenesis (**o**) in liver from ERRα^{3SA} and WT littermates fed a HFD for 17 weeks (n = 8 per group). Data are presented as means ± SEM, *p < 0.05, unpaired two-tailed Student's t test (**a**, **b**, **d-l**, **n**, **o**). Source data are provided as a Source Data file.

Supplementary Fig. 7

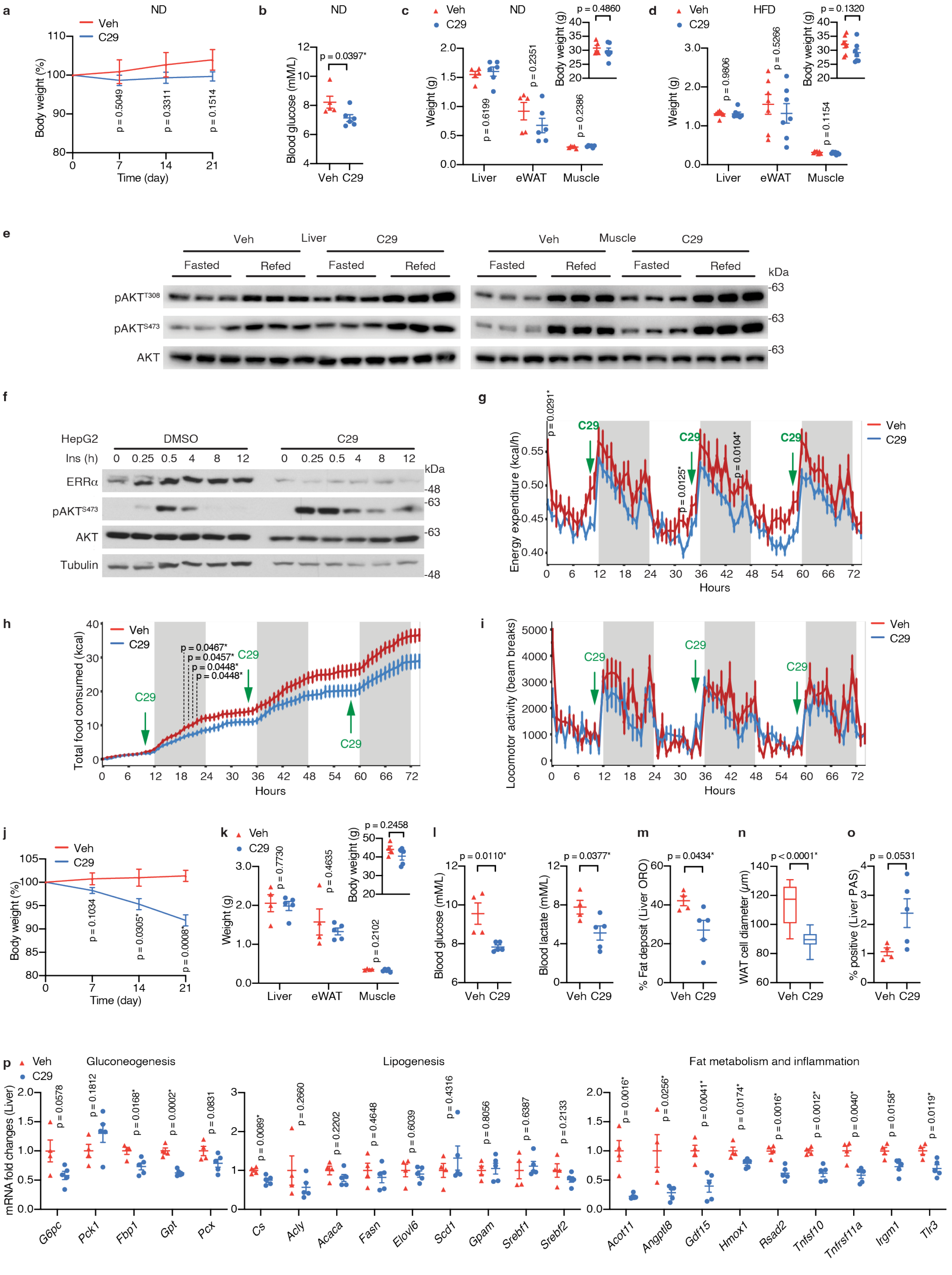

Supplementary Fig. 7 | Pharmacological ERRα inhibition improves metabolism in ERRα^{3SA} mice. a-c Analysis of the effects of 3-week C29 injections on ND-fed ERRα^{3SA} mice initiated at 12-weeks of age (Veh, n = 5; C29, n = 6). **a** Body weight changes (%) during the injections. Fed blood glucose (**b**), tissue and body weights (**c**) after 21 days of injections. **d** Tissue and body weights of HFD-fed ERRα^{3SA} mice after 21 days of injections (n = 7 per group). See also Fig. 7a. **e** Immunoblots of liver and muscle AKT phosphorylation in response to fasting and refeeding used in Fig. 7j. Each lane represents tissue from one mouse, n = 3 per group. **f**, Hepatocytes were treated with 5 μM C29 for 1 day, then serum starved for another 12 h in the presence of C29 prior to 0.58 μg/ml insulin treatment for the indicated times. **g-i** Effects of C29 on energy expenditure (**g**), total food consumed (**h**), and locomotor activity (**i**) during several days (n = 7 per group). See also Fig. 7i. **j-p** Effects of C29 on HDF-induced NAFLD (Veh, n = 4; C29, n = 5). See also Fig. 7l-r. **j** Body weight change (%) during 21 days of injection. Tissue and body weights (**k**), fed blood glucose and lactate levels (**l**) after 21 days of injections. **m-o** Quantitation of liver ORO (**m**), white adipocyte sizes quantified from four random sections per mouse (**n**), and liver PAS staining (**o**). See Fig. 7n for the stained sections. **p** Hepatic expression of genes involved in gluconeogenesis, lipogenesis, fat metabolism and inflammation. Data are represented as means ± SEM (**a-d**, **g-m**, **o**, **p**). Box plots show centre line as median, bounds of box as 25th and 75th percentiles, whiskers as minima and maxima (**n**). *$p < 0.05$, unpaired two-tailed Student's t test (**a-d**, **g-p**). Source data are provided as a Source Data file.

Supplementary Fig. 8

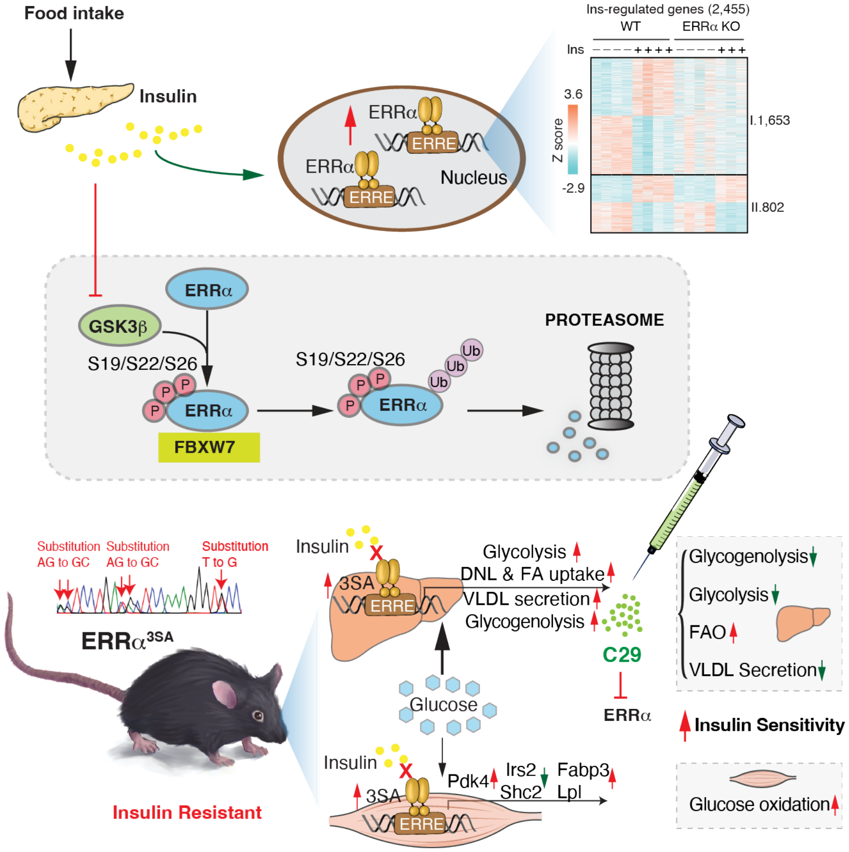

Supplementary Fig. 8 | An ERRα-dependent insulin regulatory pathway. A schematic model showing that ERRα is a potent and comprehensive insulin-responsive transcription factor. Insulin-dependent ERRα phosphorylation by GSK3β dictates ERRα stability control by FBXW7 through the proteasome. *In vivo* attenuation of this axis via genetic ablation of ERRα phosphorylation sites co-regulated by GSK3β/FBXW7 induces insulin resistance, which can be largely alleviated by ERRα pharmacological inhibitor C29.

Supplementary references

1. Zhou, Y.Y. *et al.* Metascape provides a biologist-oriented resource for the analysis of systems-level datasets. *Nat Commun* **10** (2019).

2. Hancock, M.L. *et al.* Insulin Receptor Associates with Promoters Genome-wide and Regulates Gene Expression. *Cell* **177**, 722-+ (2019).

3. Chaveroux, C. *et al.* Molecular and Genetic Crosstalks between mTOR and ERR alpha Are Key Determinants of Rapamycin-Induced Nonalcoholic Fatty Liver. *Cell Metab* **17**, 586-598 (2013).

4. Seo, Y.K. *et al.* Genome-wide analysis of SREBP-1 binding in mouse liver chromatin reveals a preference for promoter proximal binding to a new motif. *P Natl Acad Sci USA* **106**, 13765-13769 (2009).

5. Shin, D.J. *et al.* Genome-wide analysis of FoxO1 binding in hepatic chromatin: Potential involvement of FoxO1 in linking retinoid signaling to hepatic gluconeogenesis. *Nucleic Acids Res* **40**, 11499-11509 (2012).

6. Hornbeck, P.V. *et al.* PhosphoSitePlus: a comprehensive resource for investigating the structure and function of experimentally determined post-translational modifications in man and mouse. *Nucleic Acids Research* **40**, D261-D270 (2012).

7. Soltis, A.R. *et al.* Hepatic Dysfunction Caused by Consumption of a High-Fat Diet. *Cell Rep* **21**, 3317-3328 (2017).

Uncropped immunoblots for Fig. 1a, c, e, g, i, k, 2a-e, g, i, 3b, c, f-j, 4c-k, 5b, c, 7m, o and uncropped gels for Fig. 3e.

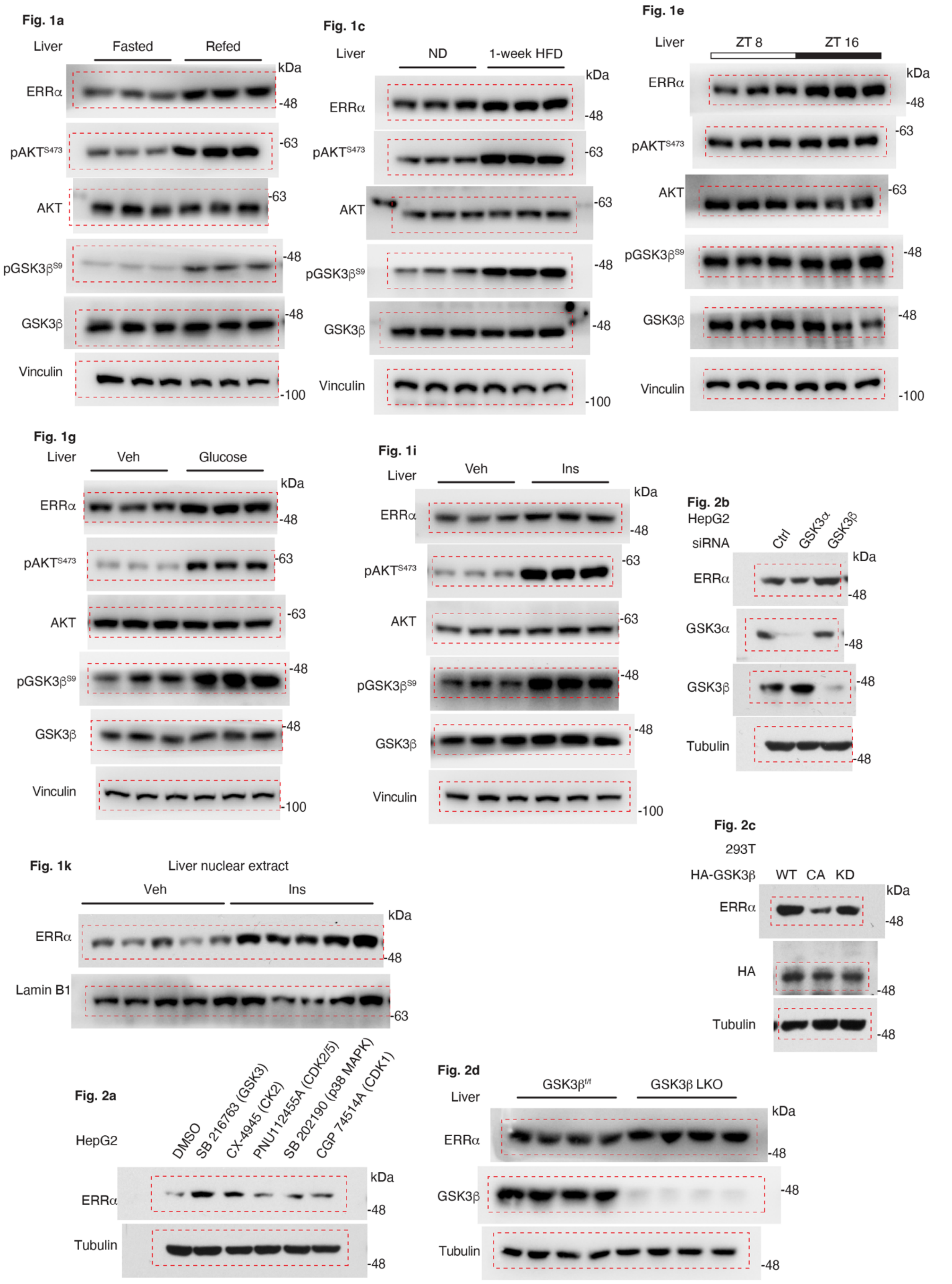

Fig. 2e

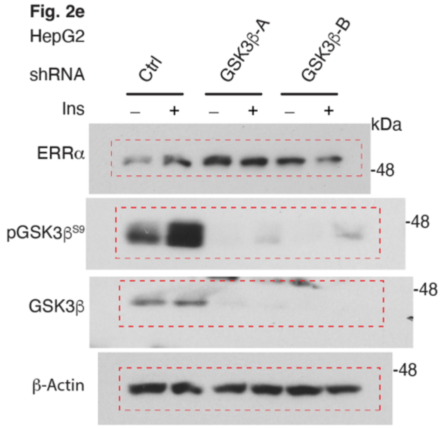

Fig. 3c

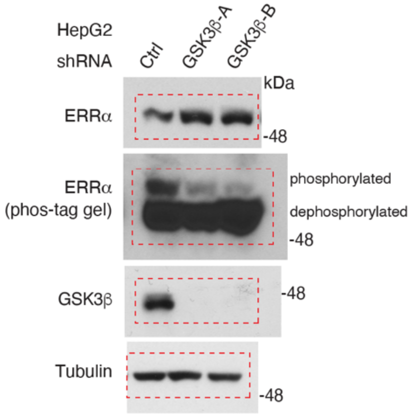

Fig. 3b

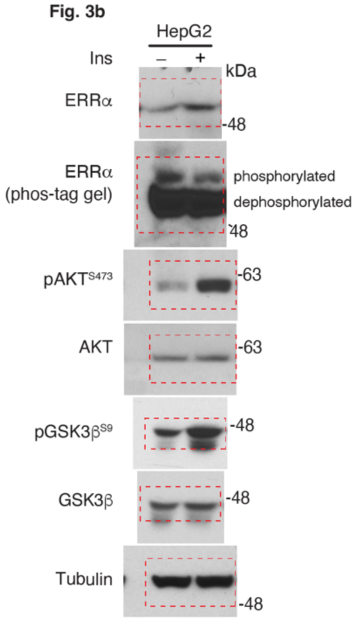

Fig. 2g

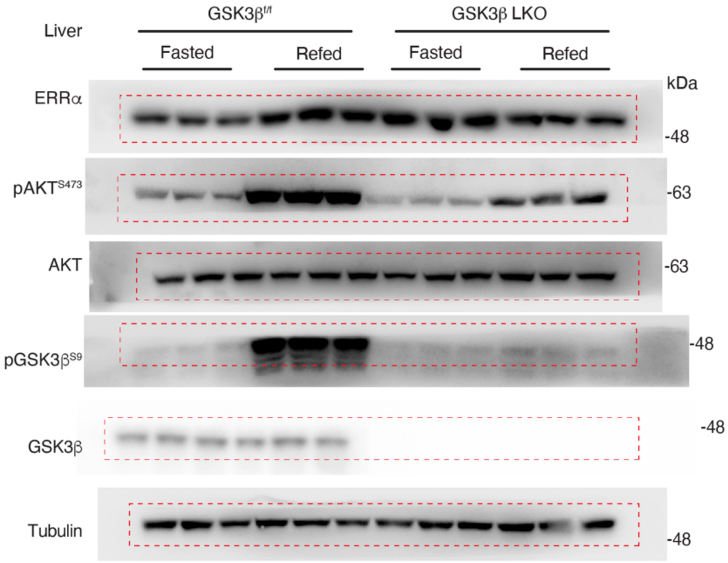

Fig. 3f

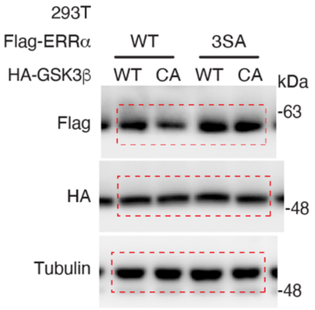

Fig. 2i

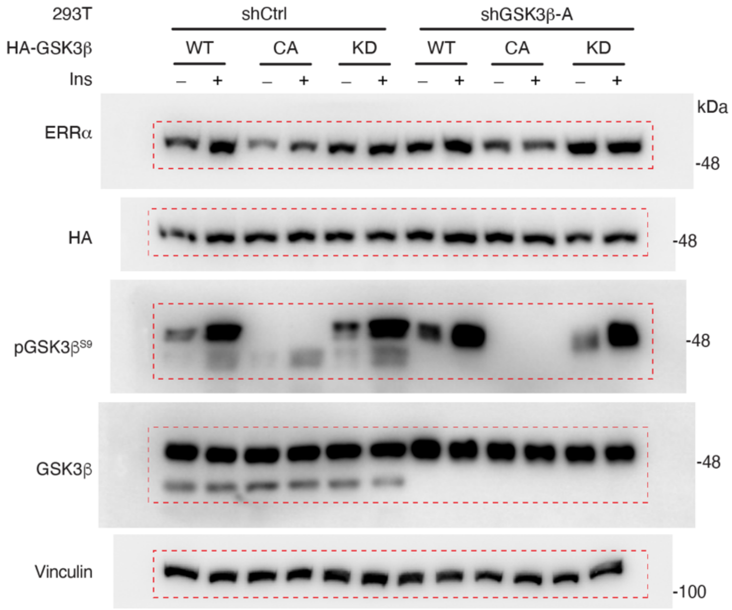

Fig. 3g

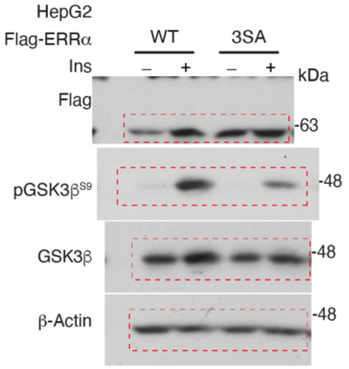

Fig. 3e

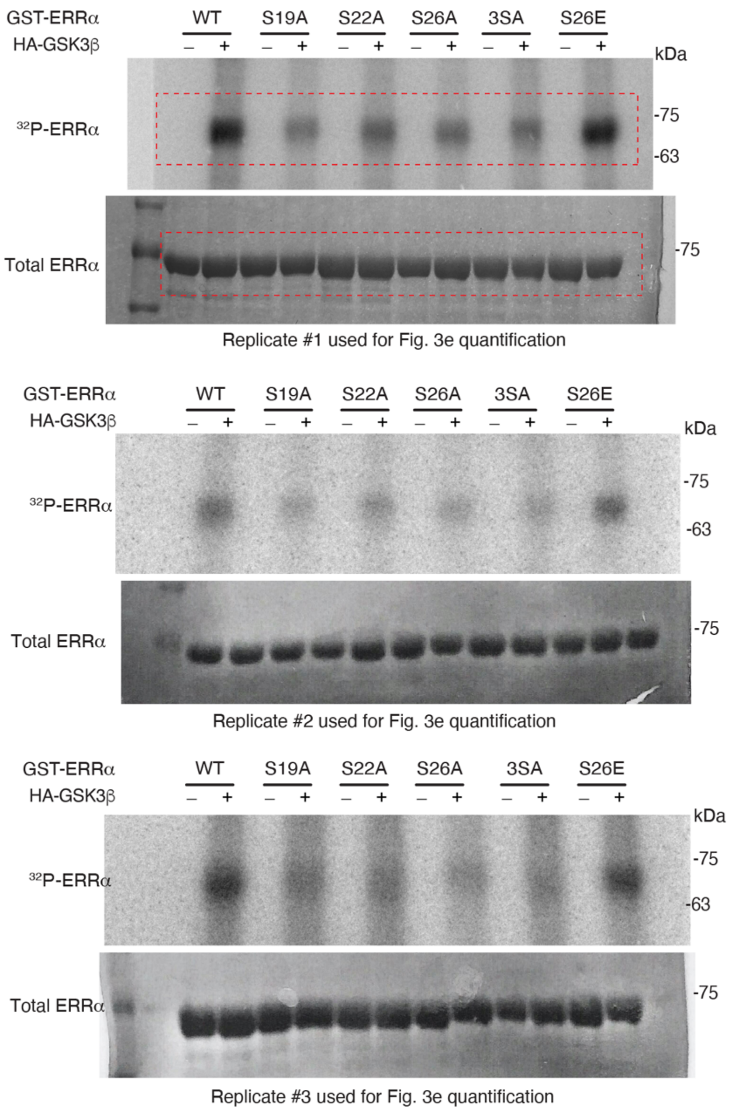

Fig. 3i

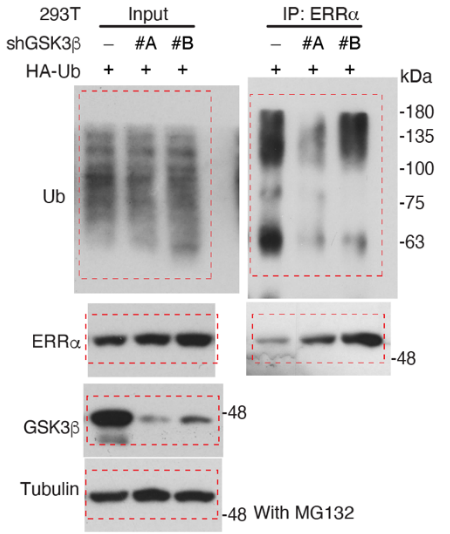

Fig. 3j

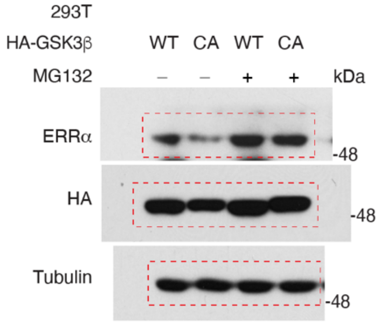

Fig. 4c

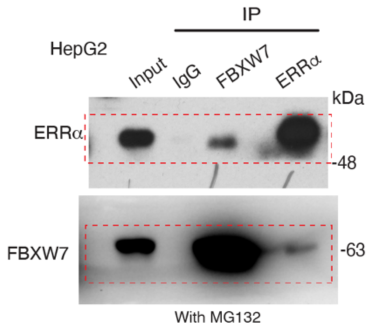

Fig. 3h

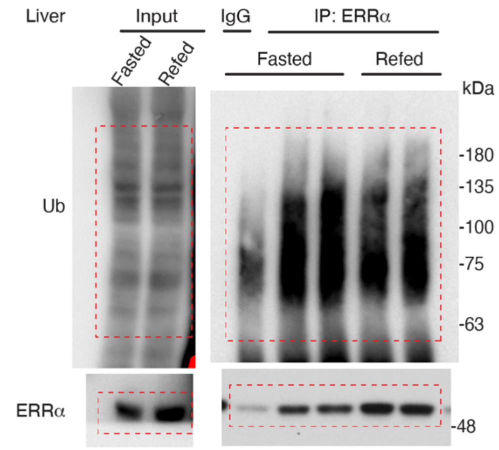

Fig. 4d

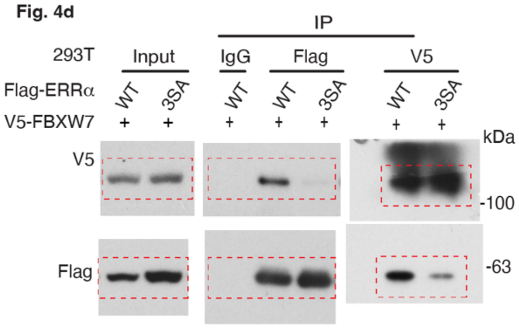

Fig. 4e

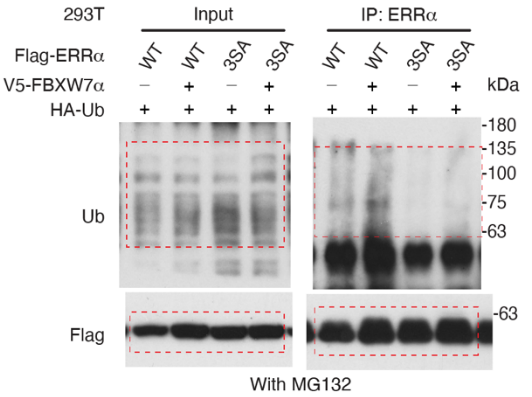

Fig. 4f

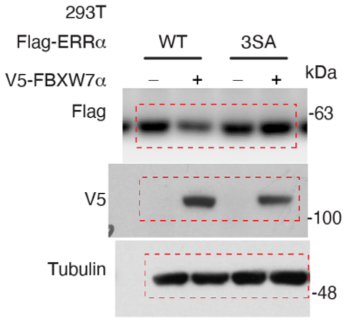

Fig. 4g

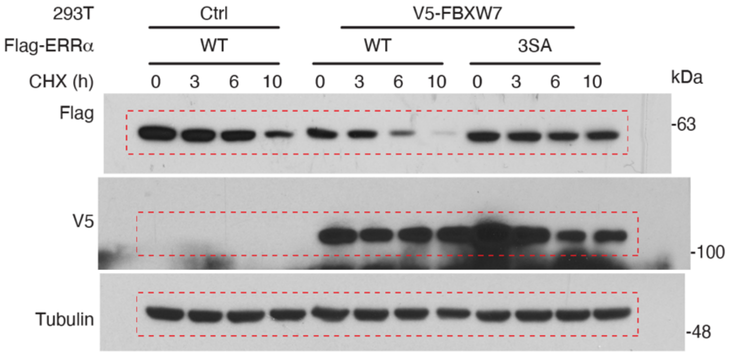

Fig. 4h

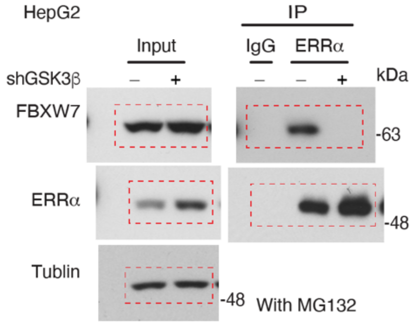

Fig. 4i

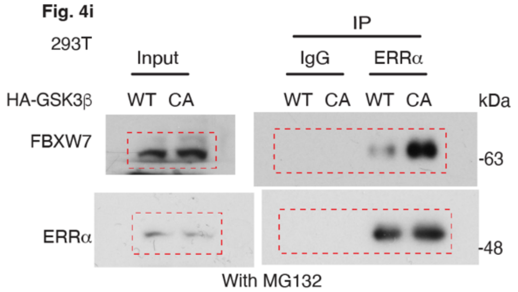

Fig. 4j

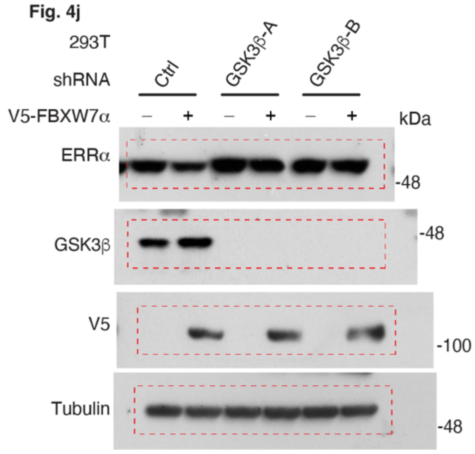

Fig. 4k

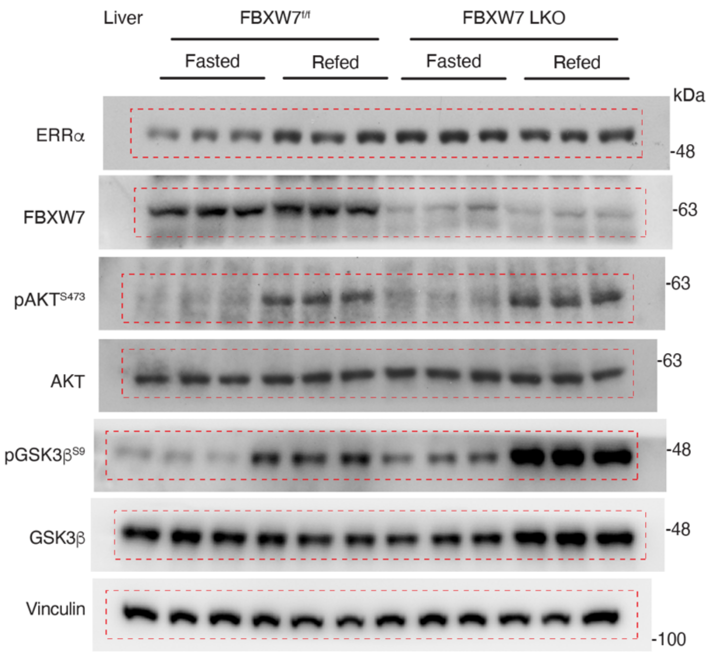

Fig. 5b

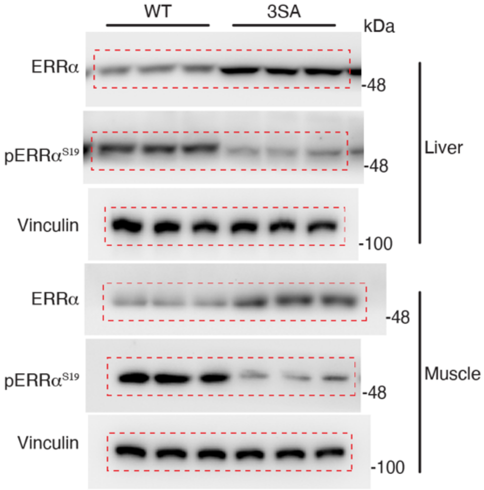

Fig. 5c

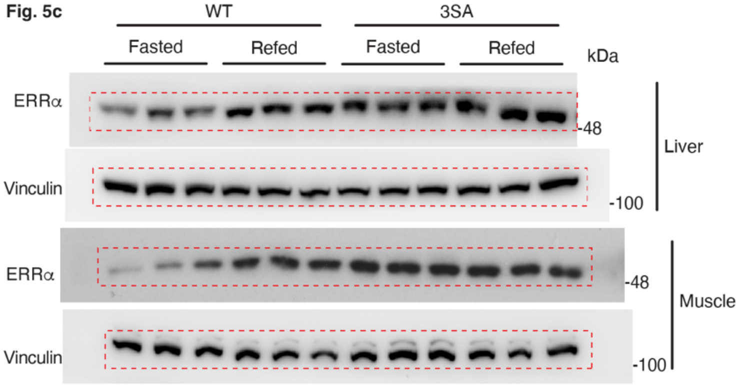

Fig. 7m

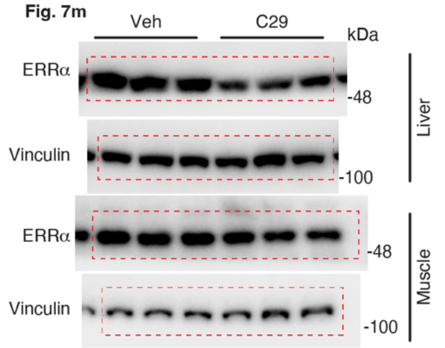

Fig. 7o

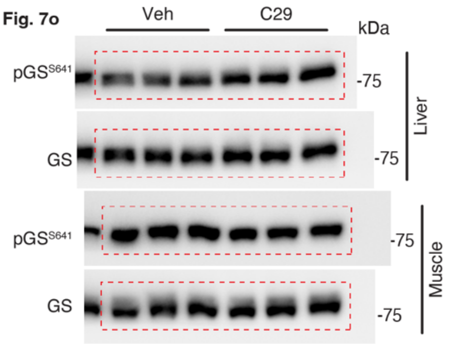

Uncropped histology staining sections for Fig. 5h, 6e, 7n.

Fig. 5h

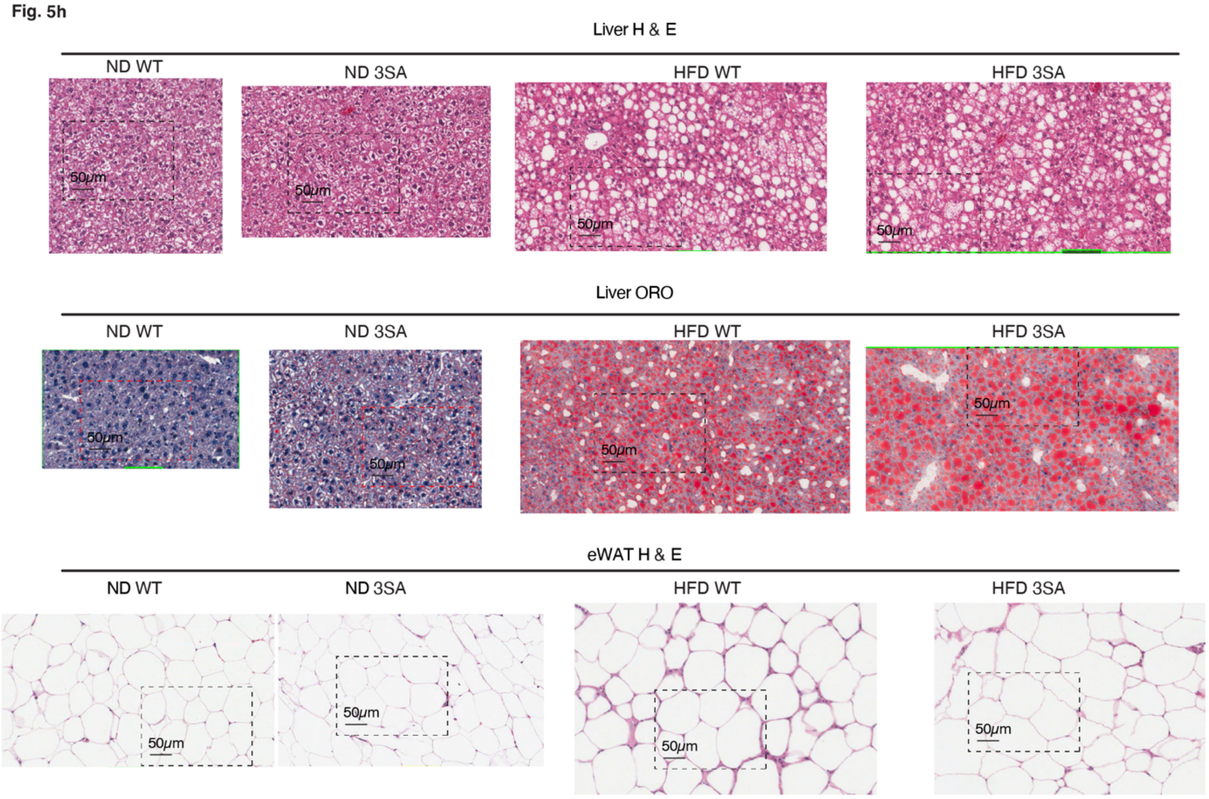

Fig. 6e

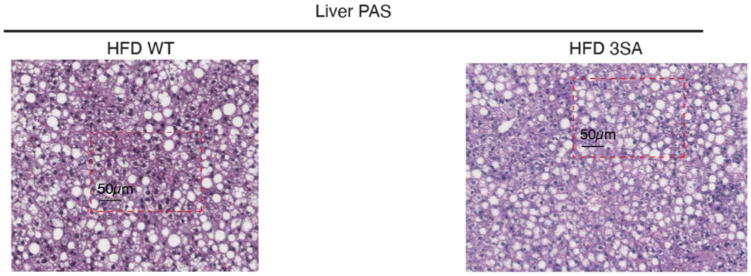

Fig. 7n

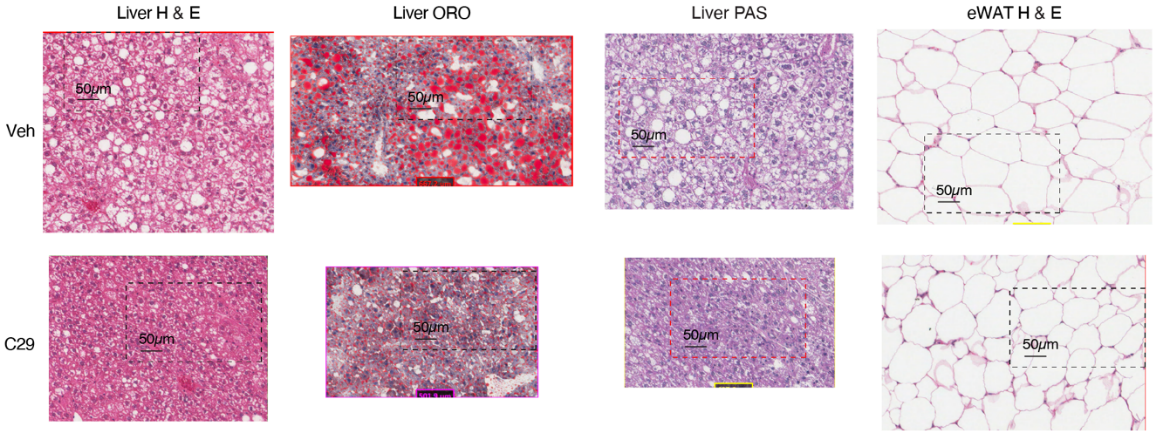

Uncropped immunoblots and gels presented in Supplementary Fig. 1a, c, e, j, 2a-e, 3a, b, d, f, 4b-l, 5b-e, h, 6c, 7e, f

Supplementary Fig. 1a

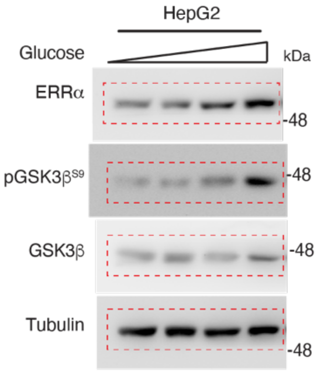

Supplementary Fig. 1c

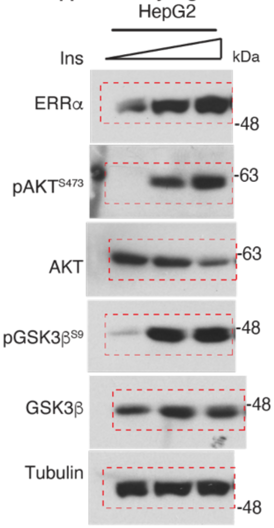

Supplementary Fig. 1e

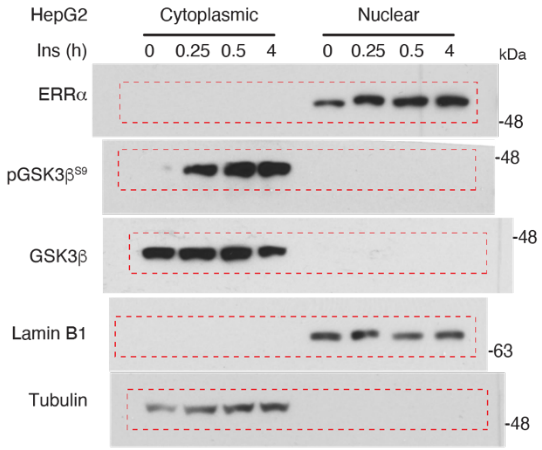

Supplementary Fig. 1j

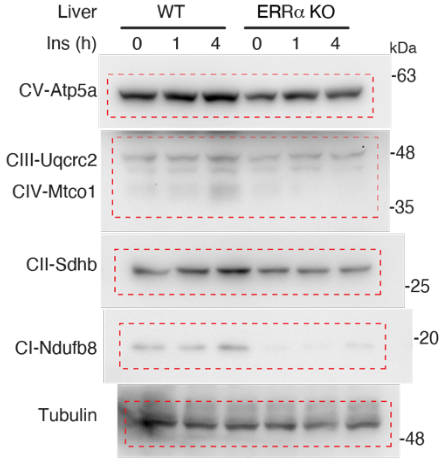

Supplementary Fig. 2a

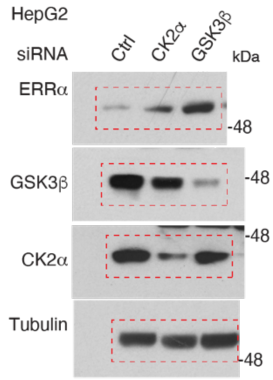

Supplementary Fig. 2b

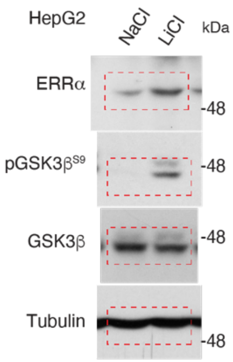

Supplementary Fig. 2c

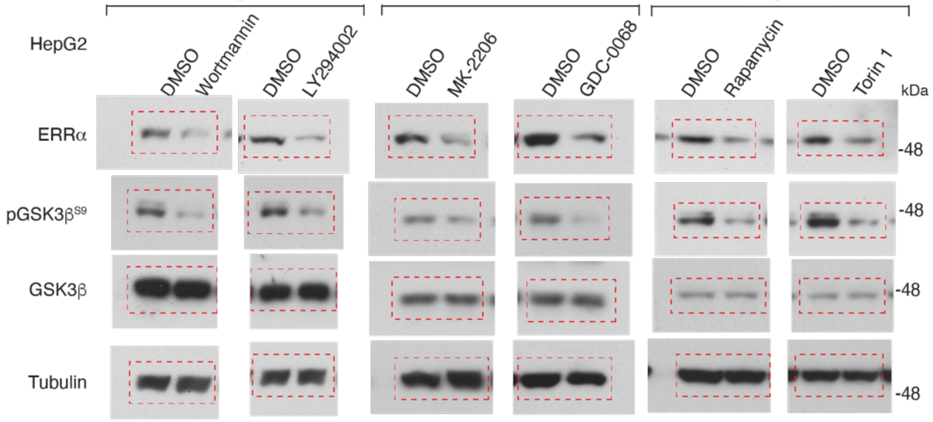

Supplementary Fig. 2d

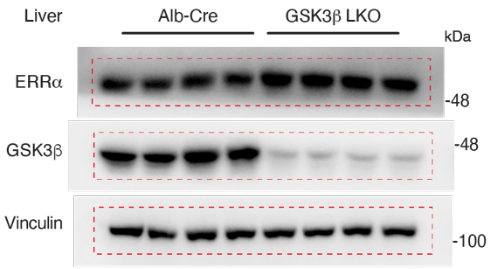

Supplementary Fig. 2e

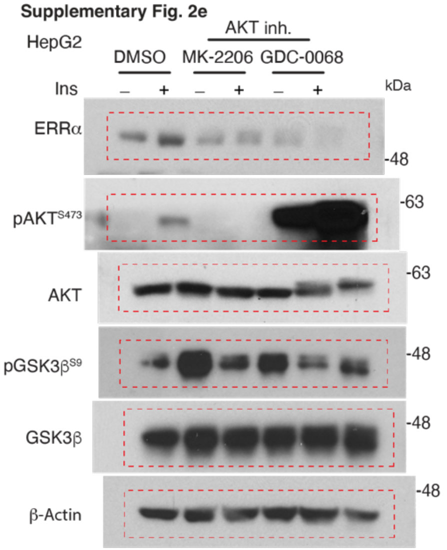

Supplementary Fig. 3b

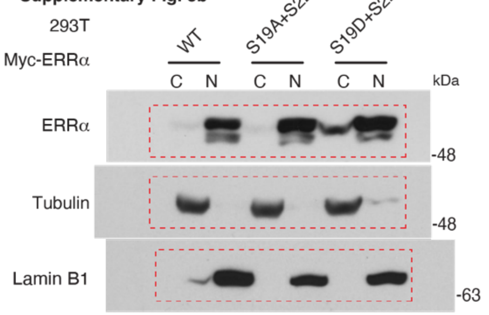

Supplementary Fig. 3d

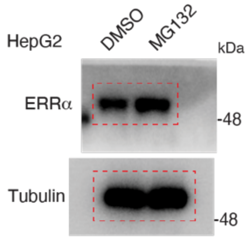

Supplementary Fig. 3f

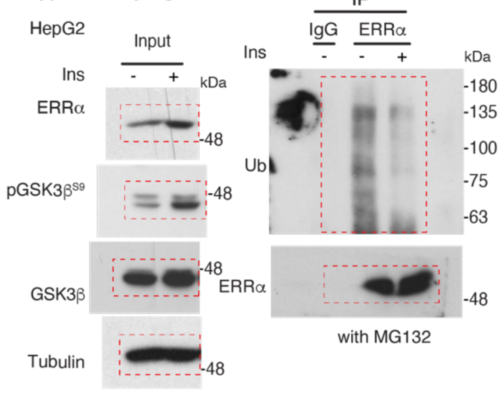

Supplementary Fig. 3a

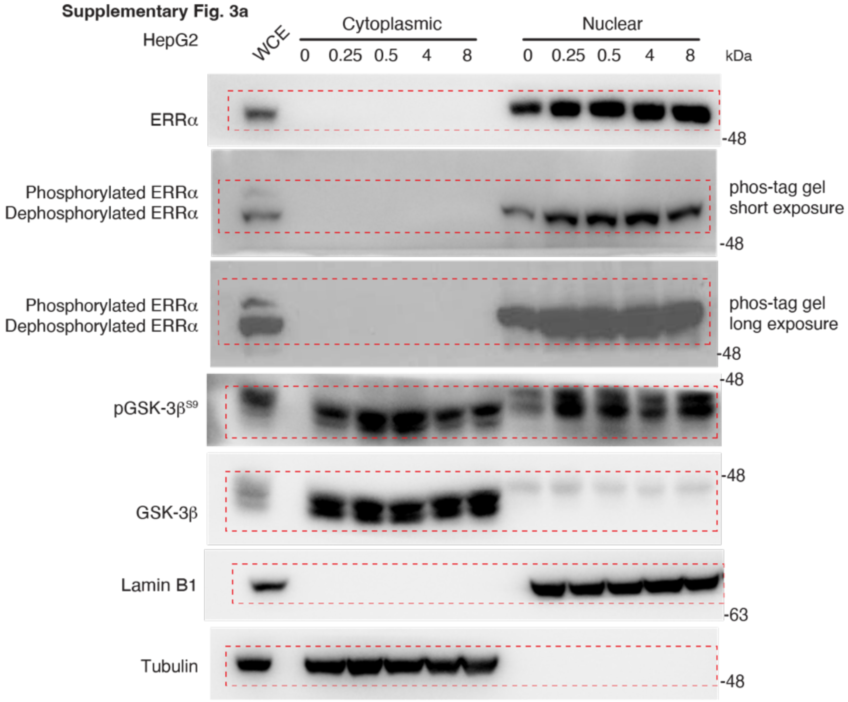

Supplementary Fig. 4b

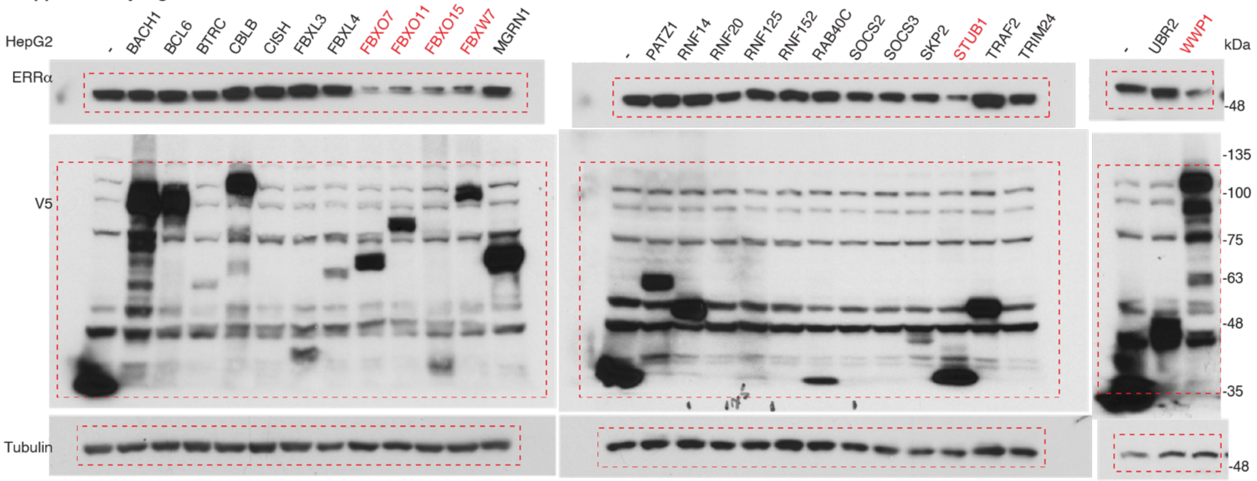

Supplementary Fig. 4c

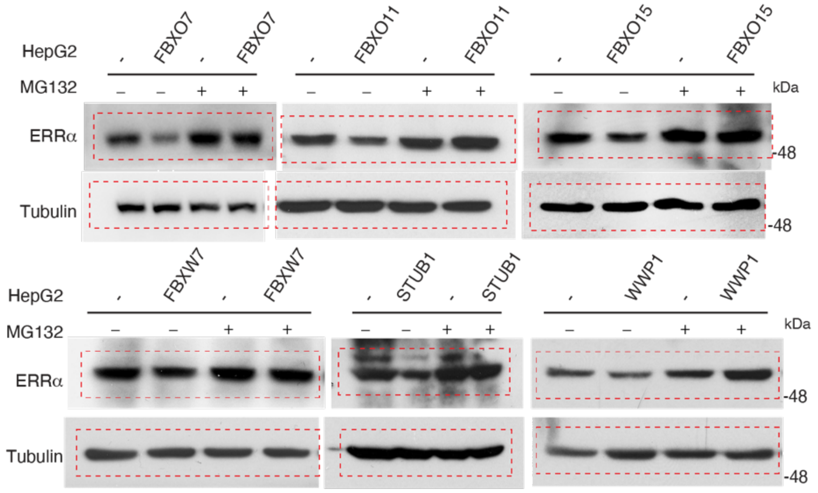

Supplementary Fig. 4d

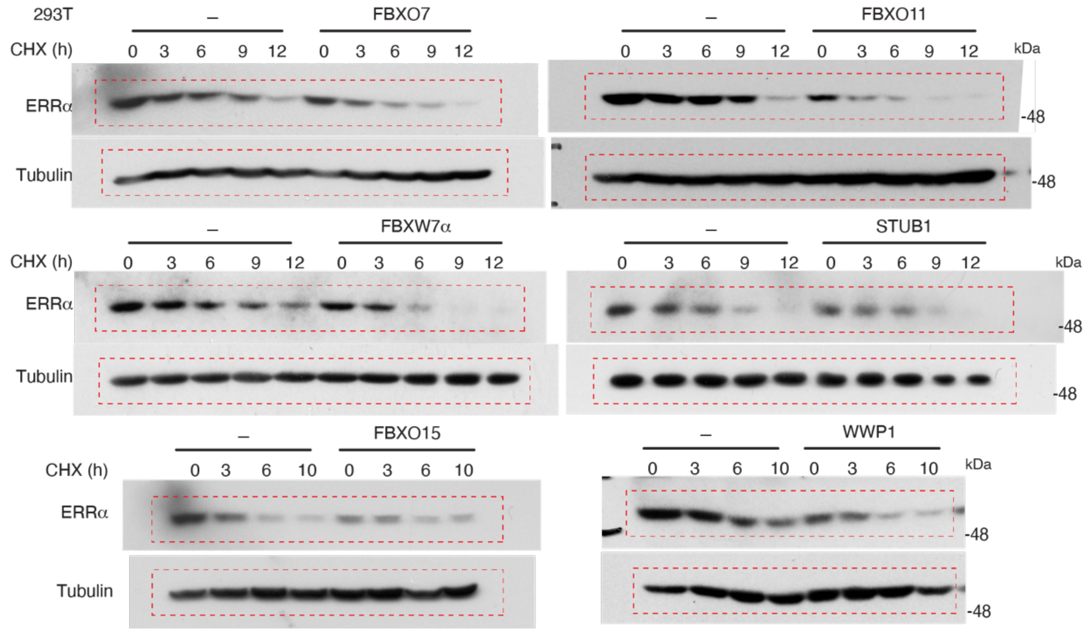

Supplementary Fig. 4e

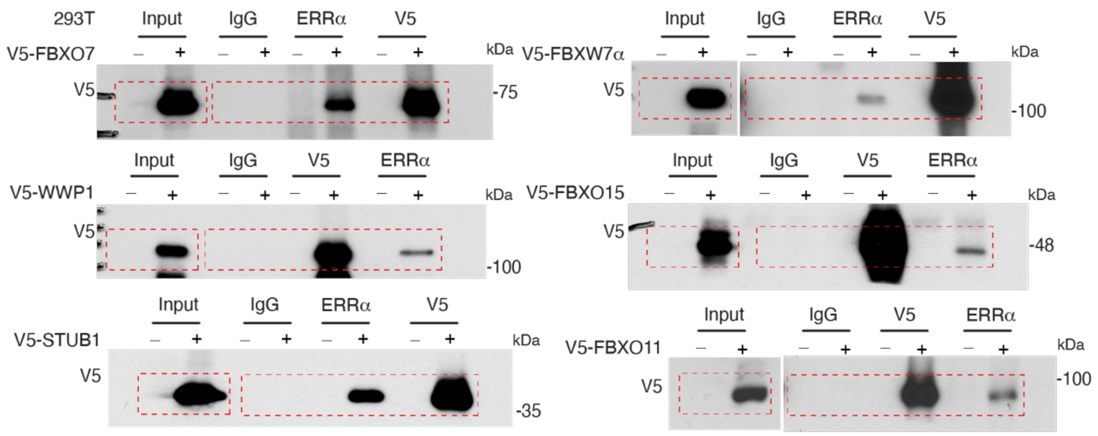

Supplementary Fig. 4g

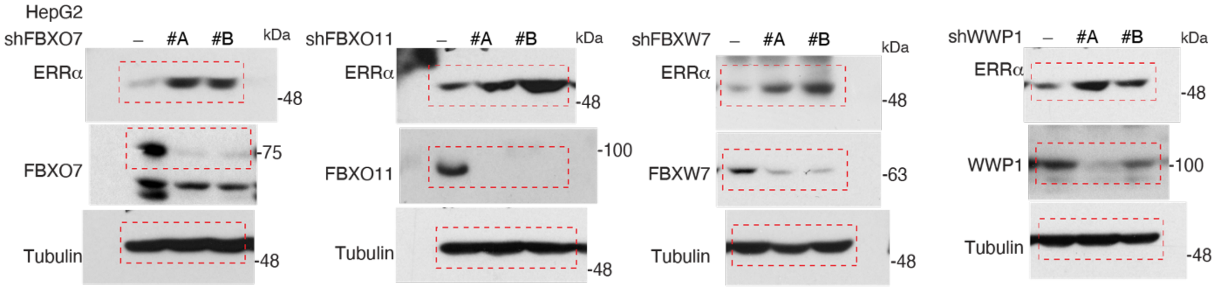

Supplementary Fig. 4f

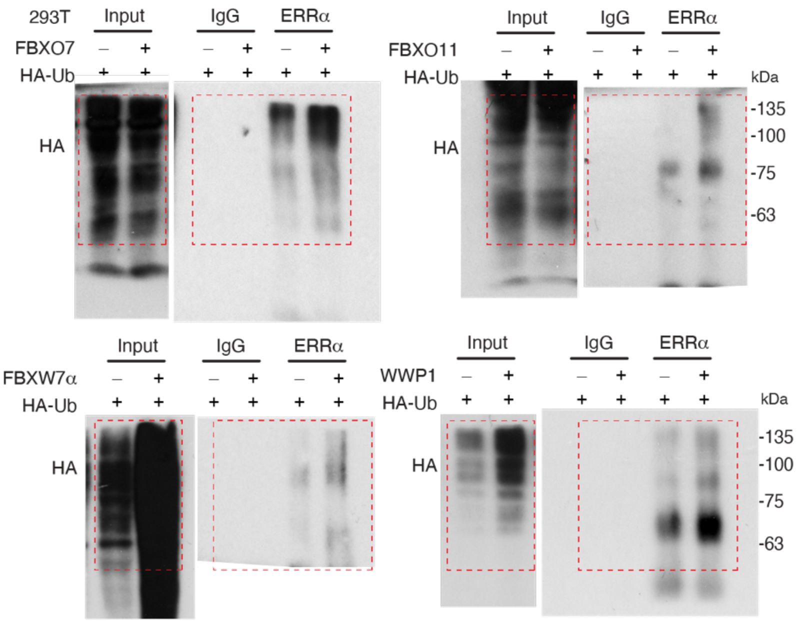

Supplementary Fig. 4i

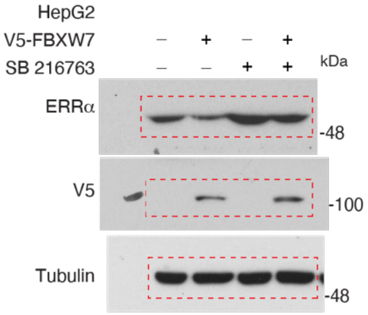

Supplementary Fig. 4j

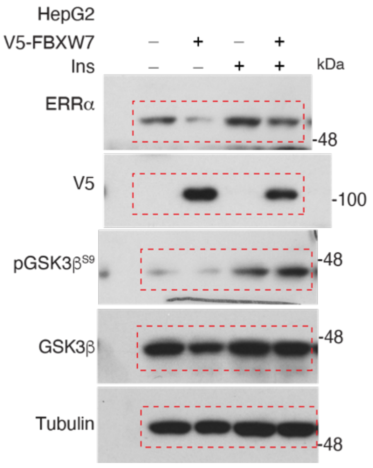

Supplementary Fig. 4h

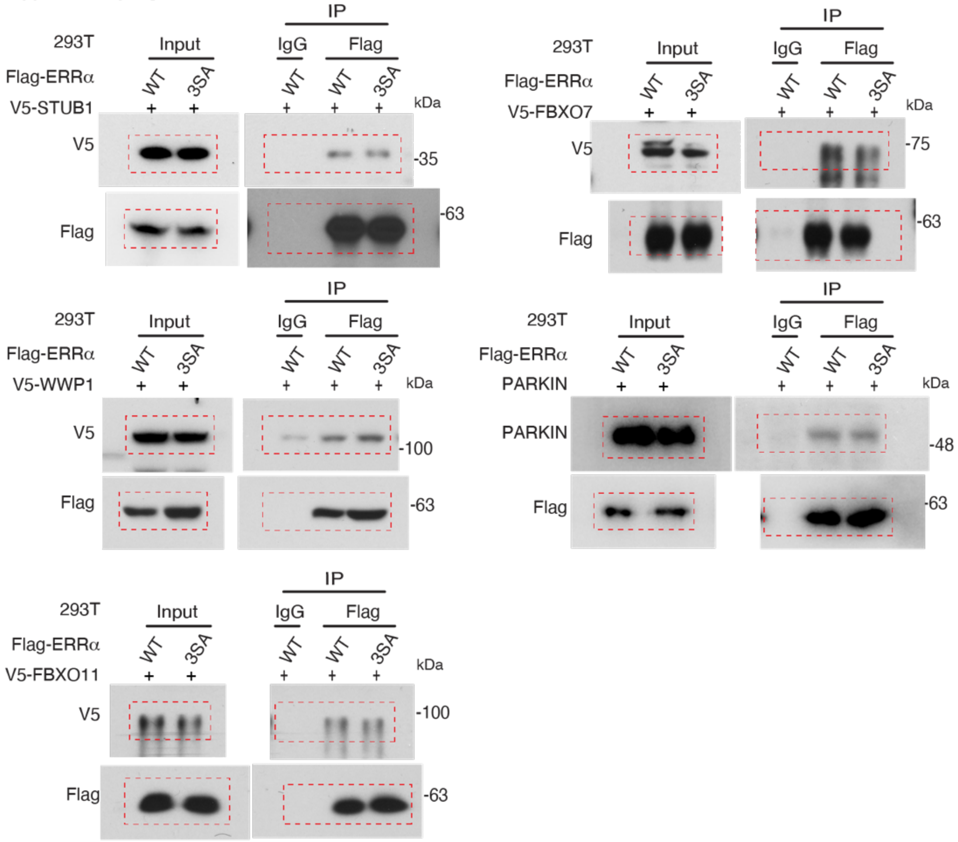

Supplementary Fig. 4k

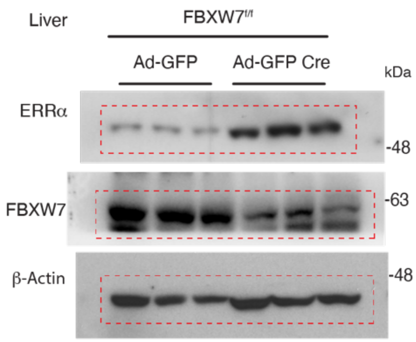

Supplementary Fig. 4l

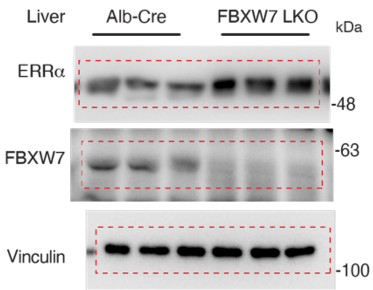

Supplementary Fig. 5b

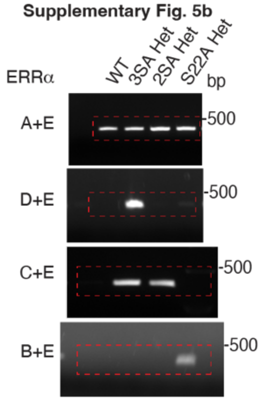

Supplementary Fig. 5c

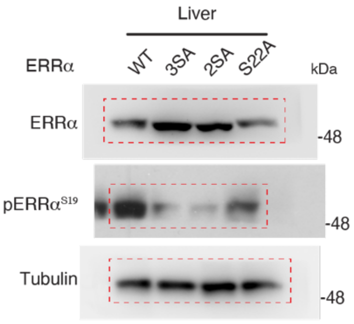

Supplementary Fig. 5d

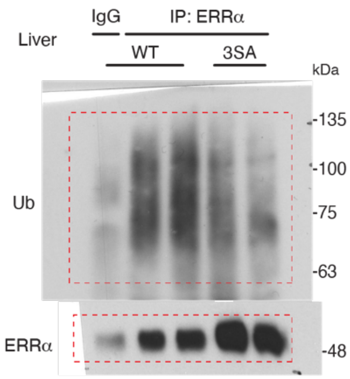

Supplementary Fig. 5e

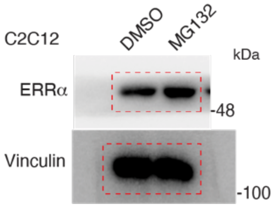

Supplementary Fig. 5h

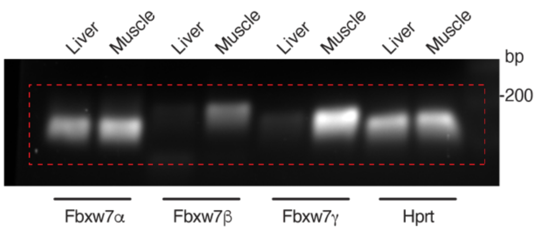

Supplementary Fig. 6c

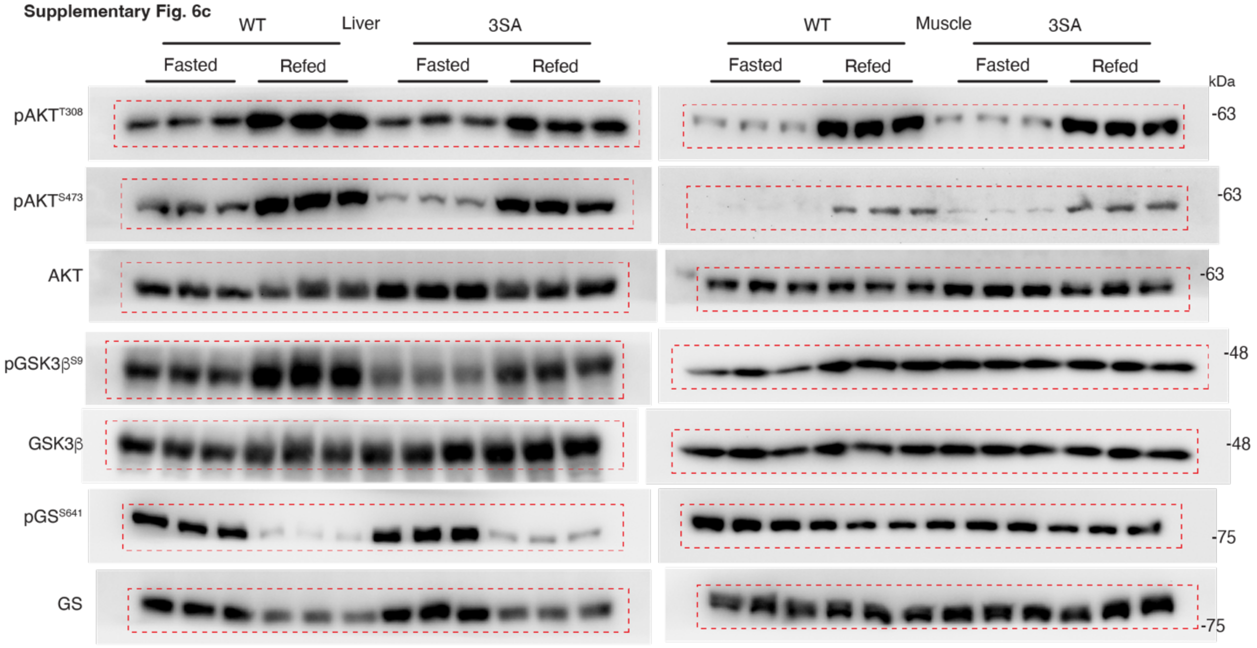

Supplementary Fig. 7e

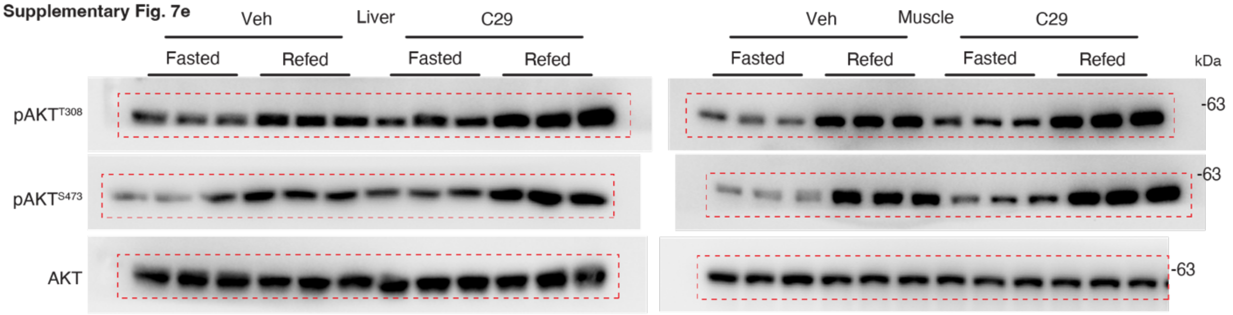

Supplementary Fig. 7f

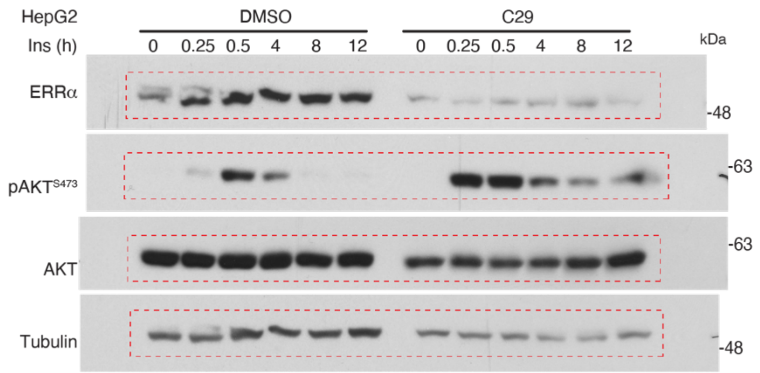

Supplement: Supplementary file 1 — Supplementary Information [file 41467_2022_29722_MOESM1_ESM.pdf]
