# Supplementary material for: Insulin action and resistance are dependent on a GSK3β-FBXW7-ERRα transcriptional axis
Source: Nat Commun. 2022 Apr 19;13:2105. doi: 10.1038/s41467-022-29722-6 (PMC9019090; doi:10.1038/s41467-022-29722-6)
Supplement: Supplementary file 4 — Reporting Summary [file 41467_2022_29722_MOESM4_ESM.pdf]

nature portfolio

Corresponding author(s): Vincent Giguere

Last updated by author(s): Mar 19, 2022

Reporting Summary

Nature Portfolio wishes to improve the reproducibility of the work that we publish. This form provides structure for consistency and transparency in reporting. For further information on Nature Portfolio policies, see our Editorial Policies and the Editorial Policy Checklist.

Statistics

For all statistical analyses, confirm that the following items are present in the figure legend, table legend, main text, or Methods section.

n/a	Confirmed	
[ ]	[x]	The exact sample size (*n*) for each experimental group/condition, given as a discrete number and unit of measurement
[ ]	[x]	A statement on whether measurements were taken from distinct samples or whether the same sample was measured repeatedly
[ ]	[x]	The statistical test(s) used AND whether they are one- or two-sided<br>*Only common tests should be described solely by name; describe more complex techniques in the Methods section.*
[x]	[ ]	A description of all covariates tested
[ ]	[x]	A description of any assumptions or corrections, such as tests of normality and adjustment for multiple comparisons
[ ]	[x]	A full description of the statistical parameters including central tendency (e.g. means) or other basic estimates (e.g. regression coefficient) AND variation (e.g. standard deviation) or associated estimates of uncertainty (e.g. confidence intervals)
[ ]	[x]	For null hypothesis testing, the test statistic (e.g. *F*, *t*, *r*) with confidence intervals, effect sizes, degrees of freedom and *P* value noted<br>*Give P values as exact values whenever suitable.*
[x]	[ ]	For Bayesian analysis, information on the choice of priors and Markov chain Monte Carlo settings
[x]	[ ]	For hierarchical and complex designs, identification of the appropriate level for tests and full reporting of outcomes
[x]	[ ]	Estimates of effect sizes (e.g. Cohen's *d*, Pearson's *r*), indicating how they were calculated

Our web collection on statistics for biologists contains articles on many of the points above.

Software and code

Policy information about availability of computer code

Data collection	1: Western blot: Epson Perfection V700 Photo (Epson) and ChemiDoc MP imaging System.<br>2: Real-Time PCR: LightCycler 480 instrument (Roche).<br>3: Kinase assay images: Typhoon TRIO Variable Mode Imager (Amersham Biosciences).<br>4: Body composition: Echo MRI-100 body composition analyser (EchoMRI LLC).<br>5: Indirect calorimetry:Sable Systems International, Promethion high-definition behavioural phenotyping system.<br>6: Histology staining images: Aperio Scanscope XT (Leica Biosystems).
Data analysis	1: Immunoblots presentation and quantification: Adobe photoshop 2021, Adobe illustrator 2021, and ImageJ (with Java 1.8.0_172).<br>2: RNA-seq analysis: STAR software (version 2.5); DESeq2 R package (version 1.14.1 and version 2_1.6.3).<br>3: Transcriptome pathway analysis: Metascape (version 3.5).<br>4: Statistics and graphing: GraphPad Prism 9.<br>5: Indirect calorimetry data analysis and graphing: CalR (version 1.3).<br>6: Histology staining images: Aperio ImageScope (version 12.4.3.5008) (Leica Biosystems).<br>7. Phosphositeplus version 6.6.0.4

For manuscripts utilizing custom algorithms or software that are central to the research but not yet described in published literature, software must be made available to editors and reviewers. We strongly encourage code deposition in a community repository (e.g. GitHub). See the Nature Portfolio guidelines for submitting code & software for further information.

Data

Policy information about availability of data

All manuscripts must include a data availability statement. This statement should provide the following information, where applicable:

- Accession codes, unique identifiers, or web links for publicly available datasets
- A description of any restrictions on data availability
- For clinical datasets or third party data, please ensure that the statement adheres to our policy

-Raw fastq file and raw gene counts of the liver RNA-sequencing data of WT and ERRα KO mice treated with saline or insulin as well as liver/muscle RNA-sequencing data of WT and ERRα3SA mice generated in this study were deposited in Gene Expression Omnibus (GEO) under the accession number GEO: GSE182000 (https://www.ncbi.nlm.nih.gov/geo/query/acc.cgi?acc=GSE182000). Reference mouse genome mm10 downloaded from the genome website browser (http://hgdownload.cse.ucsc.edu/goldenpath/mm10/bigZips/) was used for data analysis. Analyzed RNA-seq data are provided in Supplementary Data 1, 2 and 6.
-Mouse ERRα ChIP-seq GEO: GSE43638 (https://www.ncbi.nlm.nih.gov/geo/query/acc.cgi?acc=GSE43638), Liver/muscle RNA-sequencing of insulin treatment GEO: GSE117741 (https://www.ncbi.nlm.nih.gov/geo/query/acc.cgi?acc=GSE117741), RNA-sequencing of insulin treatment in HepG2 cells GEO: GSE107334 (https://www.ncbi.nlm.nih.gov/geo/query/acc.cgi?acc=GSE107334), and liver RNA-sequencing of HFD C57BL/6 mice GEO: GSE77625 (https://www.ncbi.nlm.nih.gov/geo/query/acc.cgi?acc=GSE77625) used in this study were from the original study and downloaded from GEO.
-All other data are included in Source data and Supplementary information, and Supplementary Data files with this paper. Any additional information required to reanalyze the data reported in this paper is available from the lead contact upon request.
-This paper does not report original code.

Field-specific reporting

Please select the one below that is the best fit for your research. If you are not sure, read the appropriate sections before making your selection.

☒ Life sciences ☐ Behavioural & social sciences ☐ Ecological, evolutionary & environmental sciences

For a reference copy of the document with all sections, see nature.com/documents/nr-reporting-summary-flat.pdf

Life sciences study design

All studies must disclose on these points even when the disclosure is negative.

Sample size	We did not compute statistical analyses to predetermine sample sizes before performing experiments. For in vivo study, sample size was chosen based on prior experience (PMID:14585956, PMID:29635284), and animal availability. Sample size was determined to be adequate based on the consistency of measurable differences and significance level required to attain statistical significance of p<0.05 between groups. Sample sizes for in vitro experiments (at least three independent experimental replicates) were chosen based on the standard practice of the field. Sample sizes are indicated for each experiment in the manuscript.
Data exclusions	In principle, data were only excluded for failed experiments and mice that were found to have some health condition at the time of dissection. For the measurement of serum insulin level in Fig. 6b, one 3SA fed mouse, one WT fasted mouse, and one 3SA refed mouse were excluded because of failure of getting enough high-quality serum. For the measurement of body weight in Fig. 5e, one cage (containing two WT mice and one 3SA mouse) was excluded because of severe fighting cases.
Replication	All experiments were successfully replicated in at least 2 independent experiments.
Randomization	For in vivo study, mice analyzed were age matched, littermates and weight matched whenever possible, and they were then randomly allocated to each experimental group. For in vitro experiments, groups were allocated based on the genetic background of cells and different pharmacological treatments, thus no randomization was required. Randomization is not applicable for analyzing public dataset as groups have already been determined.
Blinding	Investigators were not blinded to experimental conditions for planning of experiments due to the complexity of the experiments, e.g. western blots require samples to be loaded in appropriate orders and mice in different genotypes were measured in the rotation manner to avoid the effects caused by difference in time. Data collection on indirect calorimetry is automated and confers high objectivity. The investigators were not blinded to group allocation during analysis of indirect calorimetry data, because software CalR (Version 1.3) was used for automatic quantification and unbiased comparison of differences between genotypes.

Reporting for specific materials, systems and methods

We require information from authors about some types of materials, experimental systems and methods used in many studies. Here, indicate whether each material, system or method listed is relevant to your study. If you are not sure if a list item applies to your research, read the appropriate section before selecting a response.

Materials & experimental systems

n/a	Involved in the study	
☐	☒	Antibodies
☐	☒	Eukaryotic cell lines
☒	☐	Palaeontology and archaeology
☐	☒	Animals and other organisms
☒	☐	Human research participants
☒	☐	Clinical data
☒	☐	Dual use research of concern

Methods

n/a	Involved in the study	
☒	☐	ChIP-seq
☒	☐	Flow cytometry
☒	☐	MRI-based neuroimaging

Antibodies

Antibodies used

ERRα rabbit antibody:1:1000 dilution;Abcam;cat. no.ab76228
ERRα mouse antibody:1:1000 dilution;Novus Biologicals;cat. no.NBP2-45523;Clone OTI2C12
Phospho-AKT (Ser473) antibody:1:1000 dilution;Cell Signaling Technology;cat. no.4060S
AKT antibody:1:1000 dilution;Cell Signaling Technology;cat. no.9272S
Phospho-GSK-3β (Ser9) antibody:1:1000 dilution;Cell Signaling Technology;cat. no.9322S
GSK-3β antibody:1:1000 dilution;Cell Signaling Technology;cat. no.12456S
GSK-3α antibody:1:1000 dilution;Cell Signaling Technology;cat. no.4337S
Vinculin antibody:1:2000 dilution;Sigma-Aldrich;cat. no.MAB3574;Clone VIIF9
Lamin B1 antibody:1:2000 dilution;Cell Signaling Technology;cat. no.12586S
alpha-Tubulin antibody:1:5000 dilution;Cedarlane;cat. no.CLT9002;Clone DM1A
Total OXPHOS Rodent WB Antibody Cocktail:1:5000 dilution;Abcam;cat. no.ab110413
HA antibody:1:500 dilution, 0.5 μg/IP for IP;Santa Cruz Biotechnology;cat. no.sc-805
beta Actin antibody:1:2000 dilution;Abcam;cat. no.ab8226;Clone mAbcam 8226
CK2α antibody:1:200 dilution;Santa Cruz Biotechnology;cat. no.sc-373894;Clone E-7
Flag antibody:1:2000 dilution for WB, 0.5 μg/IP for IP;Sigma-Aldrich;cat. no.F1804;Clone M2
Ubiquitin antibody:1:1000 dilution;Cell Signaling Technology;cat. no.3933S
FBXW7 antibody:1:1000 dilution;Abcam;cat. no.ab109617
FBXW7 antibody used for studies involving FBXW7 LKO mice:1:1000 dilution;R&D Systems;cat. no.MAB7776;Clone # 800201
V5 antibody:1:2000 dilution for WB, 0.5 μg/IP for IP;Abcam;cat. no.ab9116
FBXO7 antibody:1:1000 dilution;Abnova;cat. no.H00025793-M01;Clone 4G8
FBXO11 antibody:1:500 dilution;Santa Cruz Biotechnology;cat. no.sc-393229;Clone E-9
WWP1 antibody:1:1000 dilution;LSBio;cat. no.LS C333953
Parkin antibody:1:200 dilution;Santa Cruz Biotechnology;cat. no.sc-32282;Clone PRK8
Phospho-ERRα (Ser19) antibody:1:2000 dilution;Dr. Vincent Giguère laboratory;PMID: 18063693
Phospho-Akt (Thr308) antibody:1:1000 dilution;Cell Signaling Technology;cat. no.9275S
Phospho-Glycogen Synthase (Ser641) antibody:1:2000 dilution;Cell Signaling Technology;cat. no.47043T
Glycogen Synthase antibody:1:2000 dilution;Cell Signaling Technology;cat. no.3886S
Rabbit IgG, HRP-Linked Whole Ab:1:5000 dilution;VWR;cat. no.CA95017-556L
Mouse IgG, HRP-Linked Whole Ab:1:5000 dilution;VWR;cat. no.CA95017-332L
Normal rabbit IgG:0.5 μg/IP;Invitrogen;cat. no.10500C
Normal mouse IgG:0.5 μg/IP;Invitrogen;cat. no.10400C
hERRα polyclonal antibody for IP: 1:250 dilution;Dr. Vincent Giguère laboratory;PMID: 14978033
Rabbit TrueBlot®: Anti-Rabbit IgG HRP:1:4000 dilution;ROCKLAND;cat. no.18-8816-33

Validation

ERRα rabbit antibody:Abcam;cat. no.ab76228;Manufacturer's website:https://www.abcam.com/estrogen-related-receptor-alpha-antibody-epr46y-ab76228.html
ERRα mouse antibody:Novus Biologicals;cat. no.NBP2-45523;Clone OTI2C12;Manufacturer's website:https://www.novusbio.com/PDFs2/NBP2-45523.pdf
Phospho-AKT (Ser473) antibody:Cell Signaling Technology;cat. no.4060S;Manufacturer's website:https://www.cellsignal.com/products/primary-antibodies/phospho-akt-ser473-d9e-xp-rabbit-mab/4060
AKT antibody:Cell Signaling Technology;cat. no.9272S;Manufacturer's website:https://www.cellsignal.com/products/primary-antibodies/akt-antibody/9272
Phospho-GSK-3β (Ser9) antibody:Cell Signaling Technology;cat. no.9322S;Manufacturer's website:https://www.cellsignal.com//products/primary-antibodies/phospho-gsk-3-beta-ser9-d3a4-rabbit-mab/9322
GSK-3β antibody:Cell Signaling Technology;cat. no.12456S;Manufacturer's website:https://www.cellsignal.com/products/primary-antibodies/gsk-3b-d5c5z-xp-rabbit-mab/12456
GSK-3α antibody:Cell Signaling Technology;cat. no.4337S;Manufacturer's website:https://www.cellsignal.com/products/primary-antibodies/gsk-3a-d80e6-rabbit-mab/4337
Vinculin antibody:Sigma-Aldrich;cat. no.MAB3574;Clone VIIF9;Manufacturer's website:https://www.emdmillipore.com/CA/en/product/Anti-Vinculin-clone-VIIF9-7F9,MM_NF-MAB3574-25UG
Lamin B1 antibody:Cell Signaling Technology;cat. no.12586S;Manufacturer's website:https://www.cellsignal.com/products/primary-antibodies/lamin-b1-d4q4z-rabbit-mab/12586?
utm_strategy=lev&utm_conv=mon&utm_stage=ous&utm_tactic=ppc&utm_region=hq&gclid=Cj0KCQiAkuP9BRCkARIsAKGLE8VtvgMbQUUx28nkmNiS3y1igcNtGp-MdcWDxeqkXjLqkZdygjjqZrAaAkKGEALw_wcB&gclsrc=aw.ds
alpha-Tubulin antibody:Cedarlane;cat. no.CLT9002;Clone DM1A;Manufacturer's website:https://www.biocompare.com/9776-Antibodies/91113-Mouse-AntiHuman-BetaTubulin-Monoclonal-antibody-Unconjugated-Clone-dm1a/
Total OXPHOS Rodent WB Antibody Cocktail:Abcam;cat. no.ab110413;Manufacturer's website:https://www.abcam.com/total-oxphos-rodent-wb-antibody-cocktail-ab110413.html

HA antibody:Santa Cruz Biotechnology;cat. no.sc-805;Manufacturer's website:https://datasheets.scbt.com/sc-805.pdf
beta Actin antibody:Abcam;cat. no.ab8226;Clone mAbcam 8226;Manufacturer's website:https://www.abcam.com/beta-actin-antibody-mabcam-8226-loading-control-ab8226.html
CK2α antibody:Santa Cruz Biotechnology;cat. no.sc-373894;Clone E-7;Manufacturer's website:https://www.scbt.com/p/casein-kinase-iialpha-antibody-e-7
Flag antibody:Sigma-Aldrich;cat. no.F1804;Clone M2;Manufacturer's website:https://www.sigmaaldrich.com/CA/en/product/sigma/f1804
Ubiquitin antibody:Cell Signaling Technology;cat. no.3933S;Manufacturer's website:https://www.cellsignal.com/products/primary-antibodies/ubiquitin-antibody/3933
FBXW7 antibody:Abcam;cat. no.ab109617;Manufacturer's website:https://www.abcam.com/fbxw7-antibody-ab109617.html
FBXW7 antibody used for studies involving FBXW7 LKO mice:R&D Systems;cat. no.MAB7776;Clone # 800201;Manufacturer's website:https://www.rndsystems.com/products/human-fbxw7-cdc4-antibody-800201_mab7776
V5 antibody:Abcam;cat. no.ab9116;Manufacturer's website:https://www.abcam.com/v5-tag-antibody-ab9116.html
FBXO7 antibody:Abnova;cat. no.H00025793-M01;Clone 4G8 ;Manufacturer's website:http://www.abnova.com/products/products_detail.asp?catalog_id=H00025793-M01
FBXO11 antibody:Santa Cruz Biotechnology;cat. no.sc-393229;Clone E-9;Manufacturer's website:https://www.scbt.com/p/fbxo11-antibody-e-9
WWP1 antibody:LSBio;cat. no.LS C333953;Manufacturer's website:https://www.lsbio.com/antibodies/wwp1-antibody-wb-western-ls-c333953/344312
Parkin antibody:Santa Cruz Biotechnology;cat. no.sc-32282;Clone PRK8;Manufacturer's website:https://www.scbt.com/p/parkin-antibody-prk8
Phospho-ERRα (Ser19) antibody:Dr. Vincent Giguère laboratory;PMID: 18063693;validated in ERRα phospho-mutant mice in this manuscript (Supplementary Fig. 5c)
Phospho-Akt (Thr308) antibody:Cell Signaling Technology;cat. no.9275S;Manufacturer's website:https://www.cellsignal.com/products/primary-antibodies/phospho-akt-thr308-antibody/9275
Phospho-Glycogen Synthase (Ser641) antibody:Cell Signaling Technology;cat. no.47043T;Manufacturer's website:https://www.cellsignal.com/products/primary-antibodies/phospho-glycogen-synthase-ser641-d4h1b-xp-rabbit-mab/47043?site-search-type=Products&N=4294956287&Ntt=+%28ser641%29+%28d4h1b%29+&fromPage=plp
Glycogen Synthase antibody:Cell Signaling Technology;cat. no.3886S;Manufacturer's website:https://www.cellsignal.com/products/primary-antibodies/glycogen-synthase-15b1-rabbit-mab/3886
Rabbit IgG, HRP-Linked Whole Ab:VWR;cat. no.CA95017-556L;Manufacturer's website:https://ca.vwr.com/store/product/en/16776610/anti-igg-donkey-polyclonal-antibody-hrp-horseradish-peroxidase
Mouse IgG, HRP-Linked Whole Ab:VWR;cat. no.CA95017-332L;Manufacturer's website:https://ca.vwr.com/store/product/en/16776620/anti-igg-sheep-antibody-hrp-horseradish-peroxidase
Normal rabbit IgG:Invitrogen;cat. no.10500C;Manufacturer's website:https://www.thermofisher.com/antibody/product/Rabbit-IgG-Isotype-Control/10500C
Normal mouse IgG:Invitrogen;cat. no.10400C;Manufacturer's website: https://www.thermofisher.com/antibody/product/Rabbit-IgG-Isotype-Control/10400C
hERRα polyclonal antibody for IP:Dr. Vincent Giguère laboratory;PMID: 14978033;validated in ERRα-null mice (PMID: 17488637)
Rabbit TrueBlot®: Anti-Rabbit IgG HRP:ROCKLAND;cat. no.18-8816-33;Manufacturer's website:https://rockland-inc.com/Product.aspx?id=42150

Eukaryotic cell lines

Policy information about cell lines

Cell line source(s)	HepG2, HEK293T, and C2C12 cells were from the ATCC (Manassas, VA, USA)
Authentication	Authentication of HepG2 cells was based on the expression of insulin-like growth factor II.<br>Expression of muscle-specific Myosin heavy chain protein was assessed for the C2C12 cell line.<br>HEK293T cells were not specifically authenticated beyond being obtained from ATCC.<br>All cells were checked routinely for morphology and were used at a low passage to limit potential contamination.
Mycoplasma contamination	All cells utilized were periodically tested for mycoplasma contamination using a mycoplasma PCR detection kit (cat. no. G238; Applied Biological Materials) and showed no signs of infection.
Commonly misidentified lines (See ICLAC register)	No cell lines used are listed in the database of commonly misidentified cell lines.

Animals and other organisms

Policy information about studies involving animals; ARRIVE guidelines recommended for reporting animal research

Laboratory animals	All mice experiments used age-matched male littermates (2- to 3-month-old), unless otherwise specified. Mice were housed two to five per cage at a constant environment (ambient temperature: 18°C-24°C; relative humidity: 30%-70%) under a 12-h light/dark cycle (7am-7pm light, 7pm-7am dark) with ad libitum access to water and a standard normal diet (ND; Envigo, Teklad Rodent diet 2920x; 3.1 kal/g, 24 kcal% protein, 16 kcal% fat, 60 kcal% carbohydrate) in an animal facility at McGill University. C57BL/6N ERRα KO mice were described previously (PMID: 14585956). FBXW7 floxed mice (Stock No: 017563) and GSK3β floxed mice (Stock No: 029592) on a C57BL/6J genetic background were obtained from the Jackson Laboratory and bred with Alb-Cre mice. Male C57BL/6J mice (Stock No: 000664), B6.Cg-Lepob/J mice (Stock No: 000632), and B6.BKS(D)-Leprdb/J mice (Stock No: 000697) aged 6 weeks were purchased from the Jackson Laboratory and allowed to acclimate for 2 weeks in our animal facility before tissue collection and examination. ERRα phospho-mutant mice on a C57BL/6N genetic background were generated by CRISPR/Cas9 gene editing at the McGill Integrated Core for Animal Modeling (MICAM). Transgenic lines were backcrossed with C57BL/6N mice directly purchased from Envigo to segregate alleles with potential off-targets.

Wild animals	No wild animals were used in the study.
Field-collected samples	No field collected samples were used in the study.
Ethics oversight	All mouse manipulations were performed in accordance with procedures approved by the McGill Facility Animal Care Committee within animal protocol 3173, and complied with ethical guidelines set by the Canadian Council of Animal Care.

Note that full information on the approval of the study protocol must also be provided in the manuscript.
